# Reduced expression of a subunit gene of sucrose non-fermenting 1 related kinase, *PpSnRK1βγ*, confers flat fruit abortion in peach by regulating sugar and starch metabolism

**DOI:** 10.1186/s12870-021-02850-9

**Published:** 2021-02-10

**Authors:** Jian Guo, Ke Cao, Jia-Long Yao, Cecilia Deng, Yong Li, Gengrui Zhu, Weichao Fang, Changwen Chen, Xinwei Wang, Jinlong Wu, Wenwu Guo, Lirong Wang

**Affiliations:** 1 Zhengzhou Fruit Research Institute, Chinese Academy of Agricultural Sciences, Zhengzhou, China; 2 College of Horticulture & Forestry Sciences, Huazhong Agricultural University, Wuhan, China; 3 The New Zealand Institute for Plant & Food Research Limited, Private Bag 92169, Auckland, 1142 New Zealand

**Keywords:** Flat peach, Fruit abortion, Sugar, Starch, Carbohydrate

## Abstract

**Background:**

Fruit abortion is a major limiting factor for fruit production. In flat peach, fruit abortion is present in the whole tree of some accessions during early fruit development. However, the physiological factors and genetic mechanism underlying flat fruit abortion remain largely elusive.

**Results:**

In this study, we have revealed that the fertilization process was accomplished and the reduction of sucrose and starch contents might result in flat fruit abortion. By combining association and gene expression analysis, a key candidate gene, *PpSnRK1βγ*, was identified. A 1.67-Mb inversion co-segregated with flat fruit shape altered the promoter activity of *PpSnRK1βγ*, resulting in much lower expression in aborting flat peach. Ectopic transformation in tomato and transient overexpression in peach fruit have shown that *PpSnRK1βγ* could increase sugar and starch contents. Comparative transcriptome analysis further confirmed that *PpSnRK1βγ* participated in carbohydrate metabolism. Subcellular localization found that PpSnRK1βγ was located in nucleus.

**Conclusions:**

This study provides a possible reason for flat fruit abortion and identified a critical candidate gene, *PpSnRK1βγ*, that might be responsible for flat fruit abortion in peach. The results will provide great help in peach breeding and facilitate gene identification for fruit abortion in other plant species.

**Supplementary Information:**

The online version contains supplementary material available at 10.1186/s12870-021-02850-9.

## Background

To feed the ever growing number of global human population, food security is becoming an urgent task, including fruit crop yield. Peach (*Prunus persica* L.) is the fourth economically important fruit crop in the world (FAO, http://faostat.fao.org), which is regarded as a model plant in Roseceae family due to its relatively small genome size and short juvenile phase [1]. Flat peach was first cultivated in China [2] and had been introduced to the world, which had favorite flavor and edible convenient fruit shape [3, 4]. However, the fruits aborted in some flat peach accessions during early fruit development [5–8], which has greatly limited the flat peach breeding and industry.

The flat fruit shape is controlled by a single dominant gene *S* (saucer-shaped) [9]. This gene was further mapped to the distal part of chromosome 6 using linkage analysis [5, 6] and association mapping [8, 10]. Dirlewanger et al. (2006) [6] found that some descendants from an F2 population between flat (‘Ferjalou Jalousia’) and round (‘Fantasia’) cultivars exhibited abnormal fruits which aborted soon after fruit set. This recessive character has been named *Af* and demonstrated to be linked to the flat fruit shape gene [6]. Similarly, such flat fruit abortion phenomenon was also found by other researchers and the recessive *Af* locus was supported by the segregation ratio. The very few mature fruits in the aborting flat peach tree class were also identified, which exhibited abnormal shape of fruit and seed, showing cracking fruit phenotype at the pistillar side and absent of kernel [11]. Therefore, the allele *S* linked with *af* and *s* linked with *Af*. In this case, *S/s* determines flat fruit shape and *Af/af* is responsible for flat fruit abortion trait. Recently, the key gene responsible for flat fruit shape, *PpOFP1*, has been identified and validated, and overexpression of *PpOFP1* did not result in fruit abortion in tomato and *Arabidopsis* [8, 12]. These studies indicated that flat shape and fruit abortion were genetically controlled by different genes, and the adjacent gene underling flat fruit abortion still remained unknown.

Fruit and seed abortion is a major limiting factor for achieving crop yield [13–15]. As sink organs, fruit and seed development is accompanied with the accumulation of storage products, mainly proteins, starch and oils, which are typical features of growth and maturation stages [16]. Sugars play a vital role, though many factors participate in fruit and seed set, such as source-sink interaction, hormonal signaling and other metabolic pathways [17]. Under abiotic stress, sugar limitation is a well-known factor leading to fruit and seed abortion [13, 18, 19]. In tomato, silencing gene *LIN5* which encodes an invertase resulted in fruit abortion and reduced fruit size [20]. As well, overexpression a sucrose synthase gene in cotton reduced seed abortion [21]. Therefore, genes associated with sugar metabolism might regulate fruit and seed abortion.

The sucrose non-fermenting 1 related kinases (SnRKs), also known as sucrose non-fermenting 1(SNF1) in yeast and AMP-activated protein kinase (AMPK) in mammalian, are crucial components in plant growth and development by regulating transcriptional and metabolic processes [22–24]. There are three subfamilies of SnRKs, including SnRK1s, SnRK2s and SnRK3s [25, 26]. The SnRK2 and SnRK3 subfamily are mostly involved in stress and abscisic acid (ABA) signaling [27–31], while SnRK1s regulate carbohydrate metabolism and is crucial for normal development and response to stress [32–34]. The SnRK1 exist as heterotrimeric holoenzyme, comprising a catalytic α subunit, a regulatory γ subunit, and β subunit as a scaffold linking α and γ subunits. Green plants have evolved a hybrid βγ protein to function as the γ subunit in SnRK1 complexes. The hybrid proteins also contain an N-terminal carbohydrate binding domain, that is typically only found in the β subunits. These proteins also have a more conserved γ domain and in phylogenetic analyses cluster with functional SnRK1 γ subunits from other kingdoms [35, 36].. The α subunit genes has been reported in regulating sugar and starch metabolism [37] and loss of function exhibit embryonic lethality in Arabidopsis [38]. The γ subunits, also known as SNF4 in yeast and AMPKγ in animal, are found in complexes with β subunits [22, 39]. In pea (*Pisum sativum*), SnRK1-antisense seeds have maturation defects and result in seeds abortion [40, 41]. In peach, overexpression of *PpSnRK1α* in tomato has increased sugar and starch contents [33, 34], however little is known about the function of βγ subunit in sugar metabolism, especially in regulating fruit and seed abortion.

In this study, we investigated the physiological factors for flat fruit abortion and reported a critical candidate gene, *PpSnRK1βγ,* promoting the accumulation of sugars and starch in seed and fruit, which might be responsible for flat fruit abortion in peach. These results further enriched the function of the βγ subunit of SnRK1s in carbohydrate metabolism and provided valuable genetic basis for future flat peach breeding.

## Results

### Phenotypic analysis of flat peach abortion

**Fig. 1:**
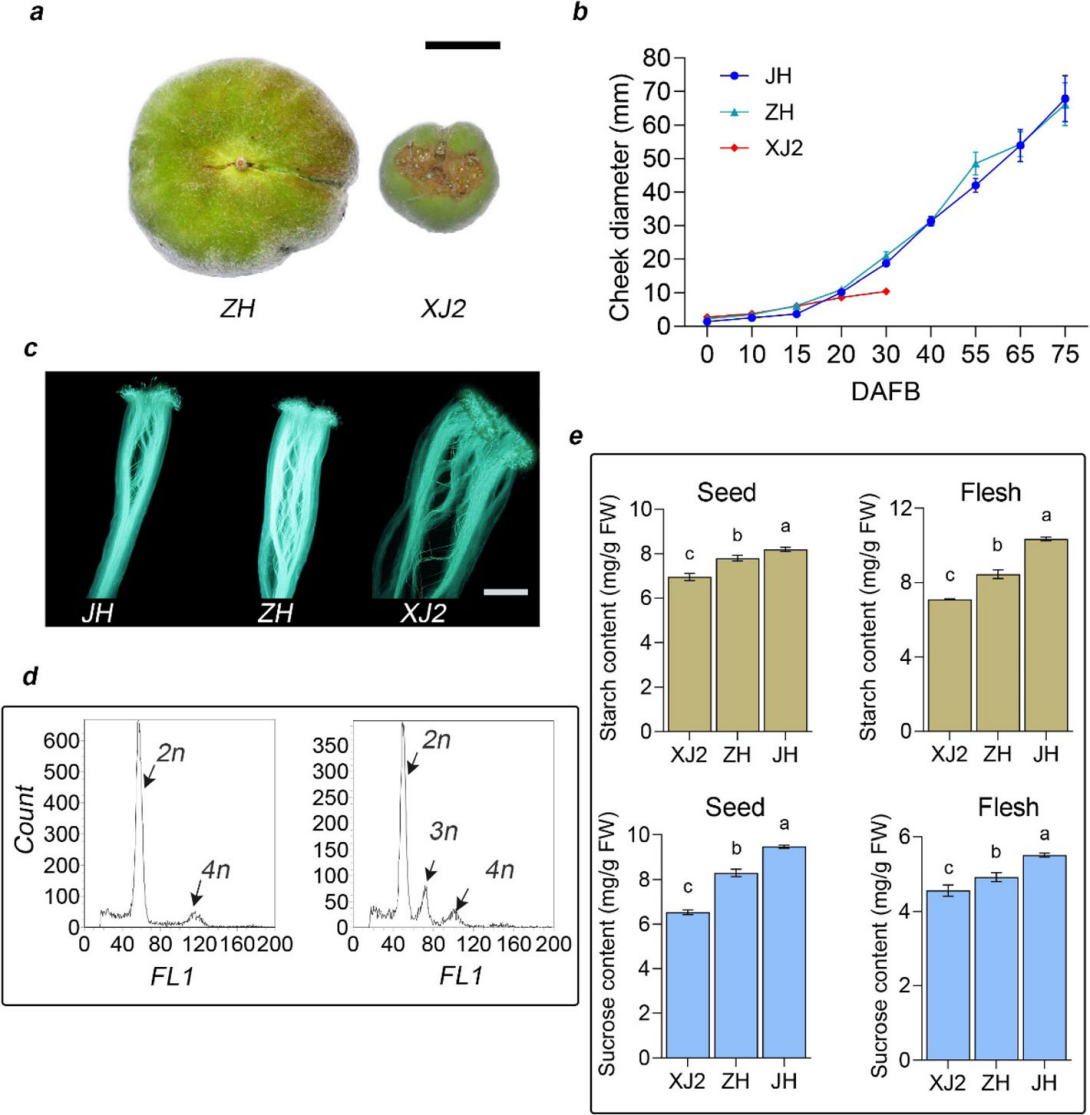
Phenotypic analysis of flat peach abortion. **a** Comparing the size of viable (‘ZH’) and aborting flat peach (‘XJ2’) at 30 DAFB. Scale bar = 1 cm. **b** Comparison of cheek diameter during fruit development among round (‘JH’), viable flat (‘ZH’) and aborting flat (‘XJ2’) peach. Bar is standard deviation (SD). **c** Pollen tube growth in ‘JH’, ‘ZH’ and ‘XJ2’. Scale bar = 1 mm. **d** Nuclear DNA content in leaves (left) and seeds (right) of aborting flat peach cultivar (‘XJ2’) was analyzed using flow-cytometry. The x axle shows DNA florescence identity peaks corresponding to the 2n, 3n and 4n nuclear DNA content of peach. The y axle shows the counts of nuclei. **e** Sucrose and starch contents in seed and fruit flesh were analyzed at 15 DAFB for ‘JH’, ‘ZH’ and ‘XJ2’. Bar is SD

The aborting flat peach cultivar was observed in our peach germplasm, showing similar phenotype with individuals obtained by Dirlewanger et al. (2006) and Picañol et al. (2012). To investigate the reason for flat peach abortion, we analyzed the phenotypic characteristics for round (‘JH’), viable flat (‘ZH’), and aborting flat fruits (‘XJ2’). Firstly, we found that the aborted flat peach stopped growing and tend to drop at 30 DAFB (days after full bloom) (Fig. 1a, b). Then, we investigated whether there was a defect in the fertilization process in aborting flat fruit, because the style size in flower organ was different from the round and viable flat peach [6]. Pollen germination and growth in pollen tube were studied, which showed that the pollen could germinate and grow into pollen tube normally (Fig. 1c). In addition, the flow cytometer checking ploidy of the fertilized embryo was carried out to determine the fertilization process. The result indicated that the fertilization process should be accomplished in ‘XJ2’, because the triploid cells were observed (Fig. 1d). Taken together, these results indicated that the fertilization process was accomplished in the aborting flat peach and there should be other factors contributing to flat fruit abortion.

It was reported that the deficiency of carbohydrate could induce seed abortion [38, 40, 41]. In this study, we found that the seed of the dropped flat peach was withered at around 30 DAFB (Figure S1). It has been reported that sugars play a vital role in fruit and seed set [17]. To investigate whether altered carbohydrate metabolism was associated with flat fruit abortion, we detected the sugar and starch content in seed and flesh for round (‘JH’), viable flat (‘ZH’), and aborting flat (‘XJ2’) peach at 15 DAFB, when all these three peach types still under healthy growth status without fruit dropping phenomenon. In this section, much lower contents of starch and sucrose in ‘XJ2’ were observed than those in viable flat and round peach in seed and fruit flesh (Fig. 1e). This observation was also consistent with their genotypes at flat fruit abortion locus, showing *af*/*af* for ‘XJ2’, *Af*/*af* for ‘ZH’ and *Af*/*Af* for ‘JH’. These results showed that the reduction of sugar and starch in seed might play vital roles in flat fruit abortion, indicating that carbohydrate metabolism participated in fruit abortion process.

### Identification and expression analysis of *PpSnRK1βγ*

**Fig. 2:**
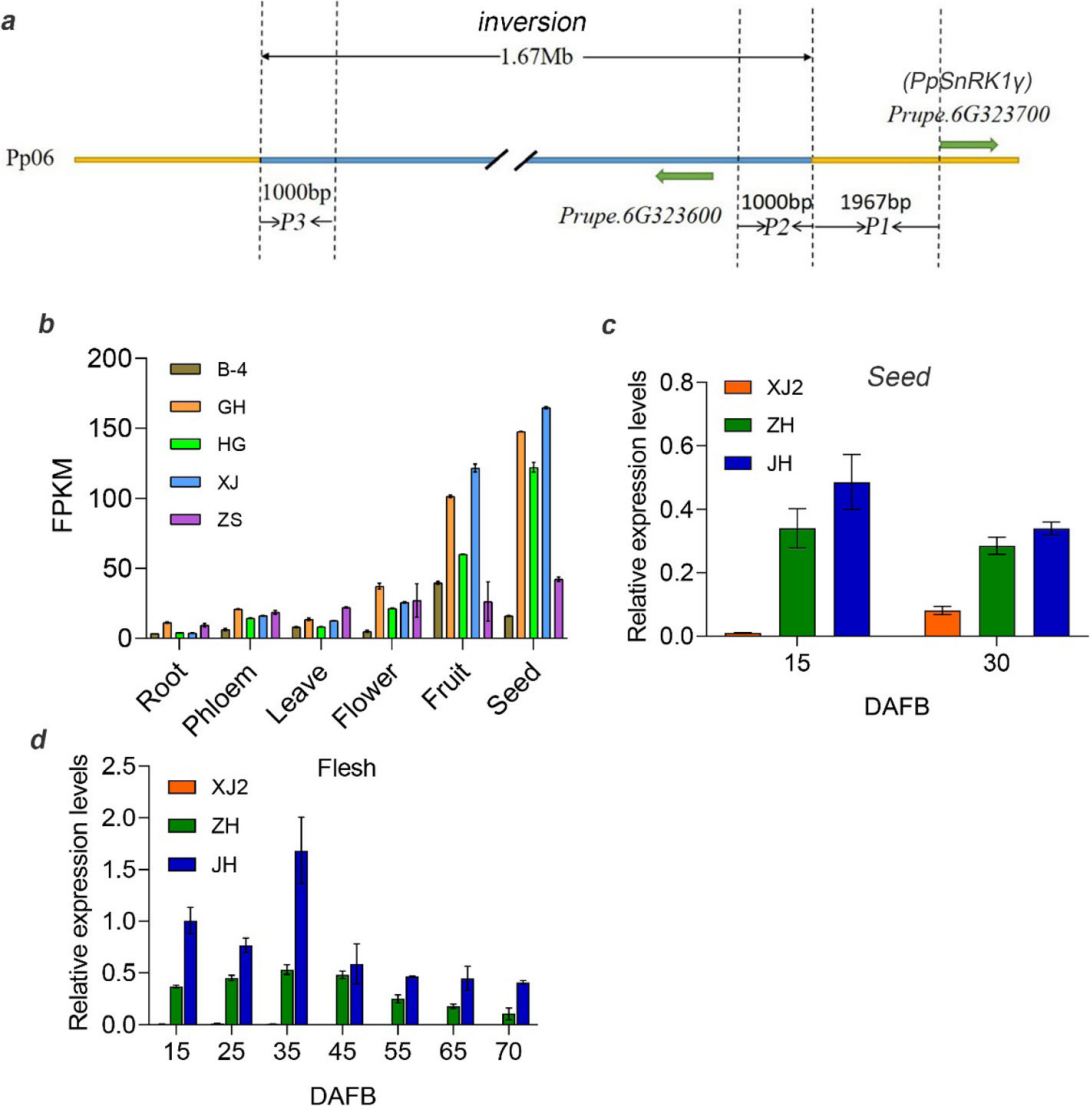
Candidate gene *PpSnRK1βγ* identification and promoter activity analysis. **a** Diagram illustrating the structure of flat fruit abortion locus. The light-blue horizontal bar indicates the 1.67-Mb inversion [8]. **b** Tissue specific analysis of *PpSnRK1βγ* using five peach accessions. **c** Gene expression analysis of *PpSnRK1βγ* in seeds among round (‘JH’), viable flat (‘ZH’) and aborting flat (‘XJ2’) peach at 15 and 30 DAFB. **d** Gene expression analysis of *PpSnRK1βγ* in flesh during fruit development. Gene expression in ‘XJ2’ was almost equal to 0, as shown beside ‘ZH’. Bar is SD

In order to elucidate the genetic basis for flat fruit abortion, candidate gene identification was carried out. We have already identified a 1.67-Mb chromosome inversion co-segregated with flat fruit shape and validated the accuracy in 336 peach accessions [8] (Fig. 2a). One key gene *PpOFP1* has been validated in controlling flat fruit shape [8, 12]. Using transcriptome data generated in our previous study [7], only 7 differentially expressed genes (DEGs) were identified in this 1.67 Mb inversion region, including *Prupe.6G303900*, *Prupe.6G311400*, *Prupe.6G313000*, *Prupe.6G314000*, *Prupe.6G314100*, *Prupe.6G317200*, and *Prupe.6G319200*. According to their genotypes, 5 of them with FPKM (fragments per kilobase of exon per million reads mapped) = 0 at some fruit developmental stages were further eliminated. One of the left two genes was annotated as unknown function protein and the other was ribonuclease, which showed no correlations with sugar and starch metabolism.

We have already analyzed the four genes around the 1.67-Mb inversion, and found that the expression levels of *Prupe.6G290900* (*PpOFP1*, flat fruit shape gene) and *Prupe.6G323700* were associated with the genotypes of flat and round peach [8]. Given that the close position between flat shape and flat fruit abortion locus, and the recessive trait, *Prupe.6G323700* was hypothesized as the candidate gene for flat fruit abortion, which was annotated as βγ subunit of SNF1-related protein kinase. In the present study, we identified the possible factors for flat fruit abortion, which was the significant reduction of sucrose and starch in seeds, but not the unaccomplished fertilization process. Tissue specific analysis showed that this gene was mostly expressed in seed and fruit (Fig. 2b). As well, gene expression was performed in seeds at 15 DAFB and 30 DAFB. The real-time PCR results showed that *Prupe.6G323700* had much lower expression in aborting flat peach than viable flat and round peach (Fig. 2c), which further indicated the reliability of this candidate gene. Gene expression pattern was consistent with their genotypes, as fruit abortion trait was recessive [6, 8]. Not only in seeds, gene expression was also performed in fruits. During fruit development, the candidate gene was much less expressed in ‘XJ2’ than in ‘ZH’ and ‘JH’, which suggested that this gene might play roles both in seeds and fruits (Fig. 2d). These results indicated that *Prupe.6G323700* might be a critical candidate gene regulating flat fruit abortion. According to its predicted protein structure and functional annotation, we named it *PpSnRK1βγ.*

### Subcellular localization of *PpSnRK1βγ*

**Fig. 3:**
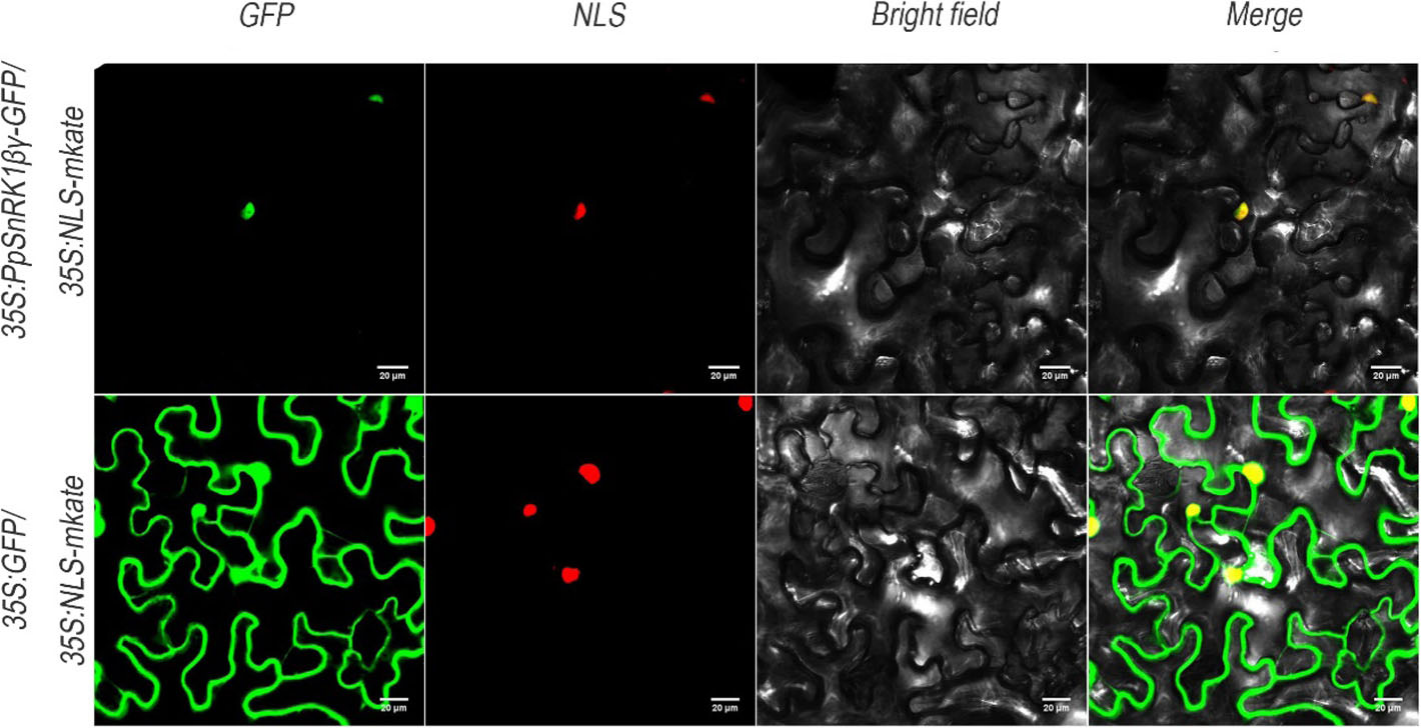
PpSnRK1βγ was located in nucleus. Mixture of Agrobacterial cells containing *35S:PpSnRK1βγ-GFP* and *35S:NLS-mkate*, or containing *35S:GFP/35S:NLS-mkate* constructs were infiltrated into tobacco leaves that were viewed using a microscope at different weave lengths for florescence of GPF and NLS-mkate, and in bright field lighting. The images taken at three different light conditions were merged. The mkate is monomeric version of katushka. Scale bar = 20 μm

To further understand the potential gene function of *PpSnRK1βγ*, subcellular localization assay was carried out. Three vectors, *35S:PpSnRK1βγ-GFP*, *35S:GFP* and *35S:NLS-makte*, were used for transient expression in tobacco (*N. benthamiana*) leaves. The *35S:NLS-mkate* was specifically localized in nucleus. The *35S:PpSnRK1βγ-GFP* and *35S:GFP* were co-transformed with *35S:NLS-mkate* respectively. In the present study, the PpSnRK1βγ-GFP fusion protein was localized in nucleus (Fig. 3), which was consistent with other research [42]. This result indicated that *PpSnRK1βγ* might play a role by affecting gene expression.

### Phylogenetic analysis of SnRK gene family

**Fig. 4:**
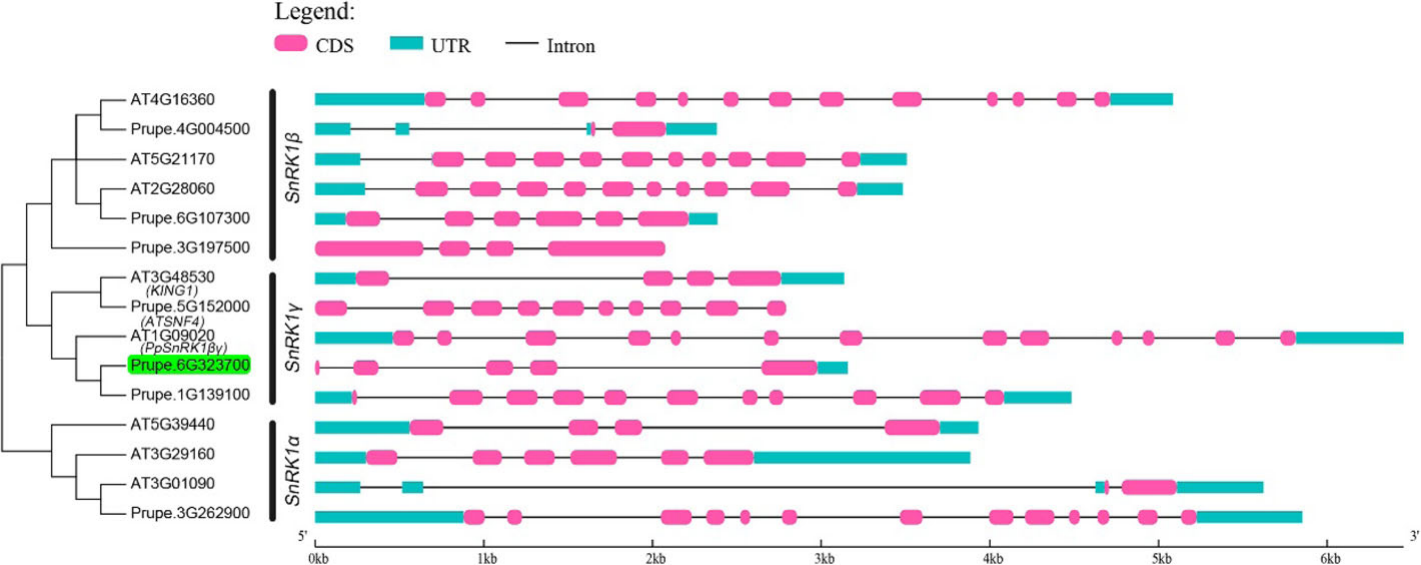
Phylogenetic tree and gene structure of SnRK1 gene family in peach and *Arabidopsis*. The referred SnRK1 sequences of *Arabidopsis* were download from NCBI and used as queries to search for homologous genes in peach. The phylogenetic tree was constructed using neighbor-joining method with bootstrap of 1000

To fully understanding the SnRK gene family in peach, genome wide gene identification was carried out. Firstly, we searched and downloaded the 43 SnRK genes in Arabidopsis from National Center for Biotechnology Information (NCBI), including three SnRK1αs, three SnRK1βs, two SnRK1γs, 10 SnRK2s, and 25 SnRK3s. We searched the similar genes in peach genome using local blast [43] and made gene annotation using Mercator [44]. As shown, one SnRK1α, three SnRK1βs, three SnRK1γs (including two SnRK1βγs), seven SnRK2s, and 17 SnRK3s were identified (Figure S2). The candidate gene identified in our study, *PpSnRK1βγ*, was clustered with *ATSNF4,* also known as *KINβγ* (Fig. 4) which had been proved to be a functional βγ subunit [42, 45].

### A chromosome inversion alters promoter activity

**Fig. 5:**
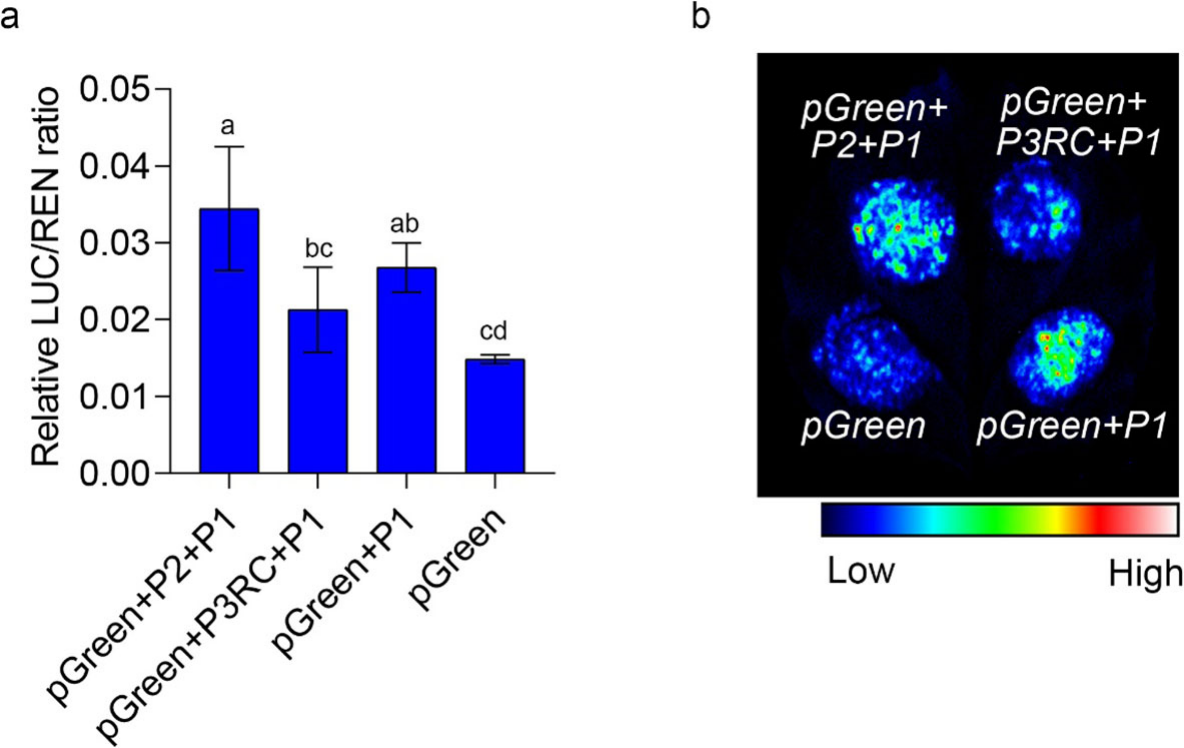
Promoter activity analysis of *PpSnRK1βγ.* **a** Relative LUC/REN ratio of promoter activity analysis. P1, P2 and P3 are fragments shown in Fig. 2c. P3RC represents the reverse complementary sequence of P3. Fragment of P2+P1 is the promoter sequence from round peach, while P3RC+P1is the promoter of flat peach. **b** Luciferase intensity indicating promoter activity. The pGreen is negative control. P1, P2 and P3RC are same as those shown in **a**. Bar is SD

To further demonstrate that *PpSnRK1βγ* expression levels were reduced by the 1.67 Mb chromosome inversion, different parts of its promoter were analyzed as shown P1, P2, P3 in Fig. 2a. These promoter fragments and their re-combinations were fused with luciferase reporter separately. Transient expressions were carried out using tobacco leaves through *Agrobacterium tumefaciens* mediated methods. As expected, the luciferase reporter gene driven by P2+P1 fragment, which was the promoter sequence from round peach, was highly expressed than the other promoter fragments, especially higher than P3RC+P1, which was the promoter of flat peach (Fig. 5a, b), indicating that this 1.67-Mb inversion reduced the promoter activity of *PpSnRK1βγ.* Furthermore, this result was verified by gene expression in ‘ZPT15’ (flat fruit shape) and its bud mutant ‘ZPT15-Mut’ (round fruit shape) peach fruits, which showed much higher expression in ‘ZPT15-Mut’ (Figure S3). All these results illustrated that the 1.67 Mb inversion reduced the promoter activity of *PpSnRK1βγ*, resulting in its much lower expression in viable and aborting flat peach than the round one.

### Overexpression of *PpSnRK1βγ* in tomato increased sugar and starch contents

**Fig. 6:**
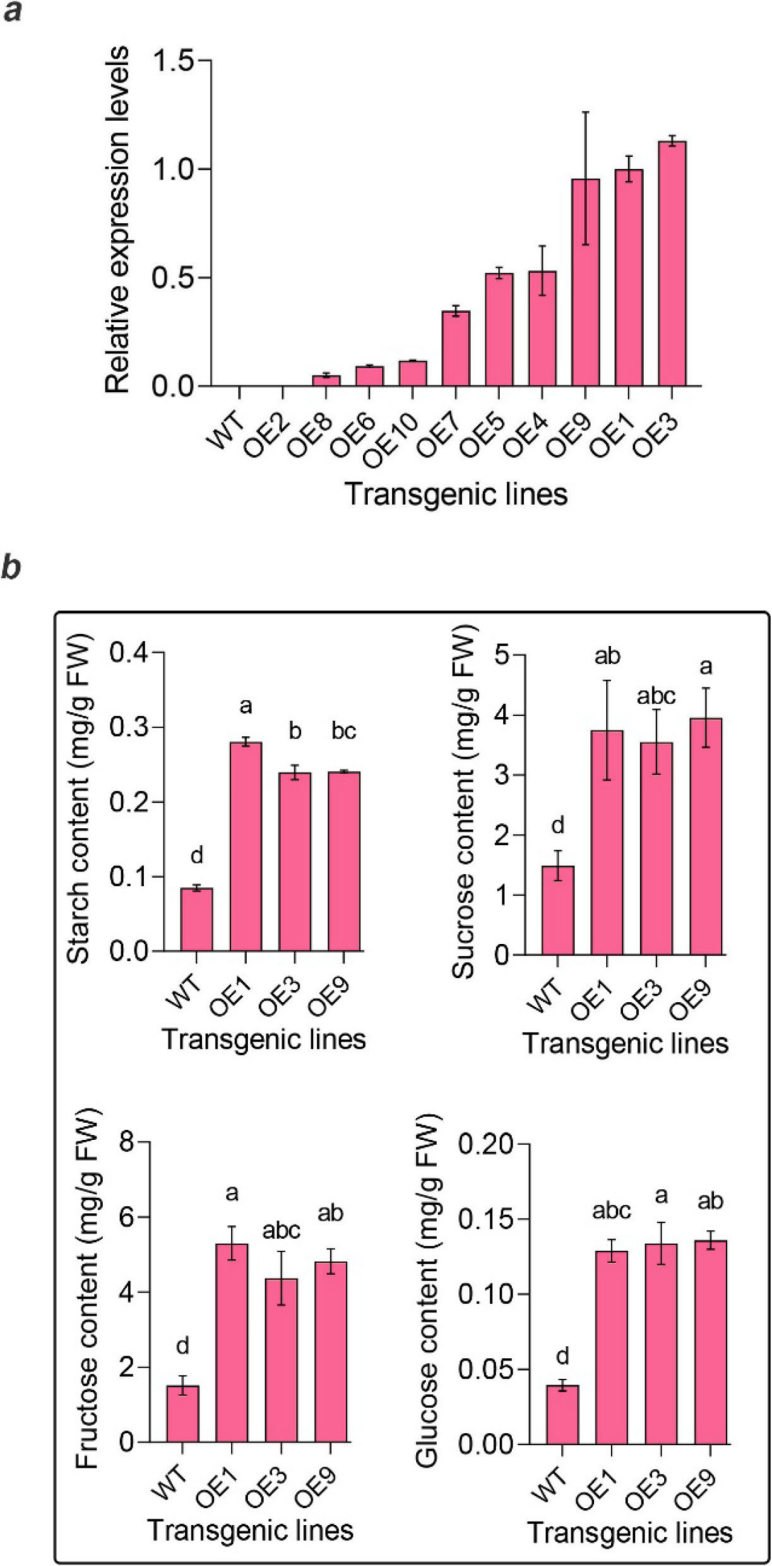
Gene expression and sugar, starch contents detection in transgenic tomato lines overexpressing *PpSnRK1βγ*. **a** Relative expression analysis of *PpSnRK1βγ* in 10 overexpression tomato lines. According to this result, we selected the top three higher expressed lines to do the downstream analysis. **b** Determination of starch, sucrose, fructose and glucose in WT and three overexpression (OE) tomato lines (OE1, OE3, OE9). Bar is SD

Up to date, the functional validation of *PpSnRK1βγ* still cannot be performed by generating stable transgenic peach lines. As an alternative approach, we used the Micro-Tom tomato genotype to validate the gene function. Ten transgenic tomato lines with different expression levels of *PpSnRK1βγ* were generated (Fig. 6a). Three of them with much higher expression levels, OE1, OE3 and OE9, were selected for sugar and starch contents determination. The results showed that the sugar and starch contents in these three OE lines were significantly higher than those in wild type (WT) (Fig. 6b) (*P* < 0.05), indicating that *PpSnRK1βγ* had function in sugar and starch metabolism.

### Carbohydrate metabolism related genes were enriched

**Fig. 7:**
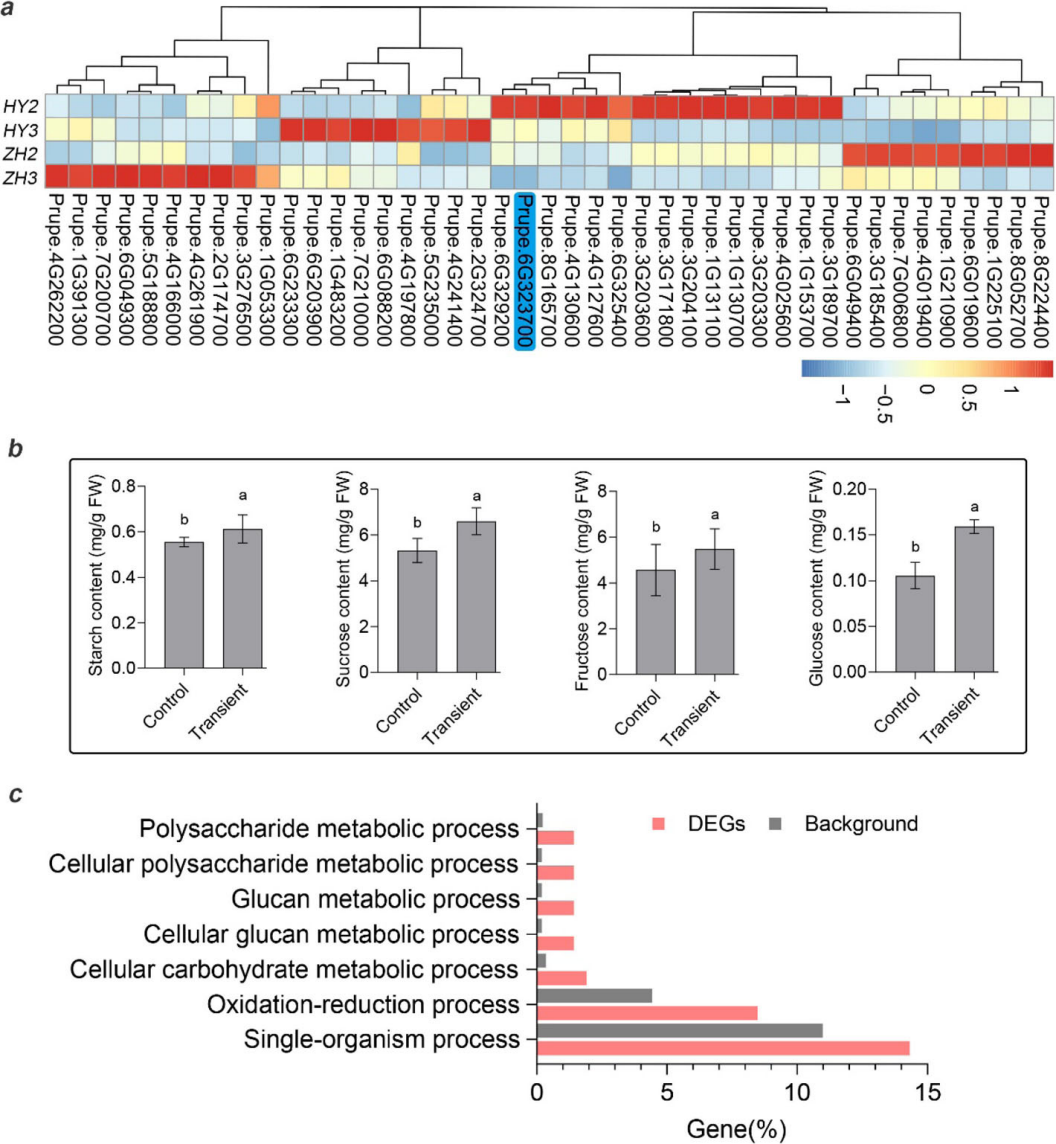
Comparative transcriptome analysis of genes participating in response to *PpSnRK1βγ*. **a** Heat map of carbohydrate related DEGs between flat and round peach during fruit development. ‘HY2’ and ‘ZH2’ stand for fruit development stage at 15 DAFB. ‘HY3’ and ‘ZH3’ is 55 DAFB. ‘HY’ is round peach and ‘ZH’ is flat. **b** Determination of starch, sucrose, fructose and glucose after transient overexpression of *PpSnRK1βγ* in peach fruit. Bar is SD. **c** GO enrichment analysis of DEGs from transient overexpression assay. ‘HY’, Zhong Tao Hong Yu (round peach); ‘ZH’, Zao Huang Pan Tao (flat peach)

During fruit development, differentially expressed genes (DEGs) were identified using transcriptome data (SRA: SRP116734) generated in our previous study [7]. In the present study, we only considered the last two developmental stages from 15 DAFB to 55 DAFB. Totally, 1350 DEGs were identified and 43 of which were related to carbohydrate metabolism. Nineteen of Forty-three were highly expressed in ‘ZH’ (flat peach) and 24 in ‘HY’ (round peach) (Fig. 7a). Considering all the 31 SnRK genes identified in peach genome, only *PpSnRK1βγ* was differentially expressed, which further suggested its role in carbohydrate metabolism during fruit development.

### Transient expression and comparative transcriptome analysis in peach

**Fig. 8:**
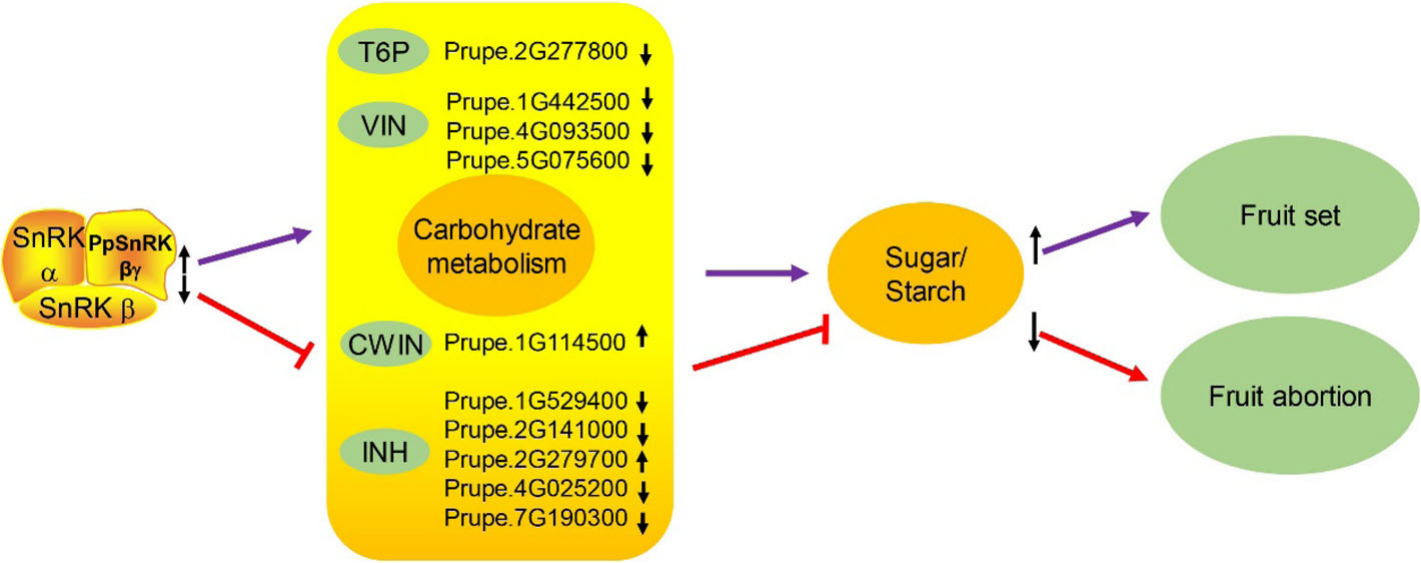
A model of *PpSnRK1βγ*-mediated flat fruit abortion in peach. Up-regulation of *PpSnRK1βγ* enhances the carbohydrate metabolism by regulating *T6P, VIN*, *CWIN*, *INH* genes et al., which result in the increase of sugar and starch contents. The sufficient sugar and starch nutrient leads to the normal fruit set. Otherwise, flat fruit would abort. T6P, trehalose-6-phosphate phosphatase; VIN, vacuolar invertase; CWIN, cell wall bound invertase; INH, invertase inhibitor

Although stable transformation in peach was limited, transient over-expression in peach fruit was executable. To validate the gene function, the 35S:*PpSnRK1βγ* was transiently expressed in ‘Hakuho’ peach cultivar at 50 DAFB using *35S:GFP* as control. After transformation, samples were collected for sugar and starch contents determination, and also used for comparative transcriptome analysis. The results showed that the sugar and starch contents were significantly increased in transient overexpression peach fruit (Fig. 7b), which further validated the gene function in carbohydrate metabolism. In addition, comparative transcriptome analysis between transient and the control samples found that 411 genes were differentially expressed and some of which were enriched in polysaccharide, glucan, carbohydrate metabolic process (Fig. 7c), including 176 up-regulated and 235 down-regulated genes (Table S1). Comparative transcriptome analysis and GO enrichment facilitated to propose a model for fruit abortion (Fig. 8). Up-regulation of *PpSnRK1βγ* affected carbohydrate metabolism by regulating *T6P* (trehalose-6-phosphate phosphatase), *VIN* (vacuolar invertase), *CWIN* (cell wall bound invertase) and *INH* (invertase inhibitor) genes, which further increased sugar and starch content in seed and fruit, resulting in fruit set. On the opposite, the flat fruit would abort (Fig. 8).

## Discussion

Fruit abortion has great influence on fruit production and food security. In peach, fruit abortion exists severely in the whole tree of some flat peach individuals. The aborting flat peach progenies were observed in a F2 population generated from flat (‘Ferjalou Jalousia’) and round (‘Fantasia’) cultivars, which could be explained either by a single dominant gene (*S/s*) or by two tightly linked gene (*S/s* and *Af/af*) [6]. It was reported that quite a few fruits could be abnormally mature with no seeds and cracking fruit phenotypes in these aborting flat peach progenies [11]. In addition, overexpression the flat shape gene, *PpOFP1*, did not result in fruit abortion in tomato [8] and *Arabidopsis* [12]. These results indicated that *PpOFP1* was the flat shape gene and the flat fruit abortion trait should be controlled by the adjacent *Af* gene locus.

To find out the casual gene responsible for fruit abortion, physiological factors were characterized. For most flowering plants, pollination and fertilization is a pre-requisite for fruit and seed set. In this study, we found that the pollination and fertilization processes were accomplished in aborting flat peach cultivar ‘XJ2’, which indicated that other processes should be involved in peach fruit abortion (Fig. 1c, d). Seed and fruit set are established soon after fertilization and transit from ovule to seed and ovary to fruit. During this process, cell division, expansion and coordinated development of seed and fruit take place immediately, including the accumulation of sugars and starch [16, 18, 46]. It has been reported that fruit and seed abortion could be induced by photoassimilate limitation in grain [47] and fruit crops [48], such as sugars and starch [13, 49]. Under optimal conditions, fruit and seed set are accomplished with sufficient nutrients, including sucrose, glucose and starch [17]. In this study, we determined the contents of sugars and starch in seeds of aborting flat, viable flat and round peach at 15 DAFB to clarify the possible reason for flat fruit abortion. The results showed that the sucrose and starch contents were significantly reduced in aborting flat fruit, which suggested that carbohydrate metabolism might be involved in fruit abortion process (Fig. 1e). Although much higher starch contents in fruits were found at 25, 35 DAFB for ‘XJ2’ (Figure S4), the reason might be that the fruit development has been already under abortion process and the underlying metabolism process was unknown. This finding was consistent with the studies on fruit and seed abortion in grain and fruit crops, and could enhance the understanding of fruit abortion in other fruit crops.

The SnRK1 subfamily comprise three subunits which play function as heterotrimeric holoenzyme, including a catalytic α subunit, a regulatory γ or βγ subunit, and β subunit as a scaffold linking α and γ subunits [35, 36]. It has been reported that the α subunit has function in regulating sugar and starch metabolism [37]. For instance, SnRK1-antisense seeds have maturation defects and result in seeds abortion by interaction of carbohydrate and hormone metabolism in pea (*Pisum sativum*) [40, 41]. As well, overexpression of *PpSnRK1α* in tomato has increased sugar and starch contents, and regulated fruit maturity and salt stress in peach [33, 34]. For β subunit, it has also been reported be involved in sugar signaling [36]. For γ subunit, it can interact with α subunit and SnRK2s [45, 50]. In *Arabidopsis*, KINβγ was a cytoplasm- and nucleus-localized protein [42], while PpSnRK1βγ was only localized in nucleus. And there are two βγ subunit genes (*Prupe.1G139100*, *PpSnRK1βγ*) in peach (Fig. 4), indicating that the other βγ subunit (Prupe.1G139100) might be localized in cytoplasm to form the SnRK1 complex. Although *KINβγ* was reported in controlling pollen development [42], hybridization between ‘JH’ (male) and ‘XJ2’ (female) was also performed in this study, which still did not alter the fruit abortion fate (data not shown). This result indicated that *PpSnRK1βγ* should play roles in other aspects but not pollen development. Little is known about the function of βγ subunit in sugar metabolism, especially in regulating fruit and seed abortion. In this study, gene function of *PpSnRK1βγ*, was validated using stable ectopic transformation in tomato and transient overexpression in peach, which both increased the sugar and starch contents (Fig. 6b, Fig. 7b). After transient overexpression of *PpSnRK1βγ* in peach fruit, 411 genes were differentially expressed and some of which were enriched in polysaccharide, glucan, carbohydrate metabolic process (Fig. 7c).

Although the expression levels of *PpSnRK1βγ* were much higher at early fruit developmental stages than those at late fruit developmental stages (Fig. 2d), the sugar contents were much higher at late fruit developmental stages (Figure S4). These results indicated that *PpSnRK1βγ* might be critical for early fruit and seed development, and could confer sugar biosynthesis in mature peach fruit together with some other related genes. It has been reported that flat peach has a more favored flavor than round peach [3, 4] and there was one soluble solid content (SSC) QTL around the fruit shape locus [51, 52]. By evaluating the SSC for 12 flat and 28 round peaches, we confirmed that the SSC in flat peach was much higher than that in round peach (Figure S5a). Hence, to make sure whether *PpSnRK1βγ* could confer a more favored flavor in flat peach fruit, its expression was analyzed at fruit maturation stage, which found that the flat peach cultivars had much higher expression levels than the round ones (Figure S5b). In addition, we used another two cultivars to perform gene expression during fruit developmental stage, which showed much higher expression in flat peach (‘ZPT10’) than round peach (‘HY’) at fruit maturation stage (Figure S6). Much higher expression of *PpSnRK1βγ* at fruit maturation stage might explain why the flat peach has much better flavor than round one. However, the opposite result of *PpSnRK1βγ* activation in flat peaches at fruit ripening stage still need to be well explored in the future.

## Conclusions

In this study, the physiological factors and genetic basis for flat peach abortion were investigated. we have revealed that the fertilization process was accomplished and the reduction of sucrose and starch contents might result in flat fruit abortion. A key candidate gene, *PpSnRK1βγ*, was identified by integrating gene expression analysis, comparative transcriptome analysis and gene transformation. A 1.67-Mb inversion co-segregated with flat fruit shape altered the promoter activity of *PpSnRK1βγ*, resulting in much lower expression in aborting flat peach. Ectopic transformation in tomato and transient overexpression in peach fruit have shown that *PpSnRK1βγ* could increase sugar and starch contents. Comparative transcriptome analysis further confirmed that *PpSnRK1βγ* participated in carbohydrate metabolism. This study provides a possible reason for flat fruit abortion and identified a critical candidate gene, *PpSnRK1βγ*, that might be responsible for flat fruit abortion in peach. The results will provide great help in peach breeding and facilitate gene identification for fruit abortion in other plant species.

## Methods

### Plant materials and sample collection

Three peach cultivars were used in this study, including one round peach ‘Zhong Nong Jin Hui’ (‘JH’), one viable flat peach ‘Zao Huang Pan Tao’ (‘ZH’) and one aborting flat peach ‘Xinjiang Pan Tao 2#’ (‘XJ2’) for which the fruit aborted at around 30 DAFB. ‘JH’ and ‘ZH’ have similar developmental period. We collected fruit samples at 0, 7, 15, 30, 40, 55, 65 DAFB for sugar and starch content measurements, check diameter measurement and gene expression analyses. Six fruits per stage were measured for the check diameter. Fruit flesh and seeds were separated at 15 DAFB, when developmental seeds were visible and could be stripped out. All cultivars used in this study were grown in the field of National Horticulture Germplasm Resources Center at Zhengzhou Fruit Research Institute, Chinese Academy of Agricultural Sciences (34°42’N, 113°42’E), Henan Province, China.

### Pollen tube elongation

Flowers of ‘JH’, ‘ZH’ and ‘XJ2’ were emasculated and pollinated with pollen collected from ‘Zhong Tao Hong Yu’ (‘HY’). At 12 h after pollination, the pistils were collected and incubated in a solution of acetic acid: ethanol at a 1:9 ratio [53] for overnight. The pistils were then passed through an ethanol series (90, 70 and 50%) and water (three times) at 3-mins intervals separately before they were immersed in 1 M NaOH for overnight and stained with 0.1% (w/v) aniline blue dissolved by 0.1 M K_3_PO_4_ in dark for than 1 h. Stained pollen tubes in the pistils were observed using an epi-fluorescence microscope fitted with a camera (DP71; Olympus).

### Ploidy detection using flow cytometry

Nuclei were isolated from young leaves and 15-day-old seeds of ‘XJ2’, and run through a flow-cytometer (PARTEC, Germany) after they were stained with DAPI solution (PARTEC, HR-B) following a protocol reported by Guo et al. [54]. Three replicates were carried out in this part.

### RNA extraction and gene expression analysis

The total RNA was extracted from the three cultivars during fruit development using an RNA extraction kit (Waryong, China). First-strand cDNA was synthesized from 0.5 μg of total RNA in a 10-μL reaction volume by ReverTra Ace qPCR RT Master Mix with gDNA Remover (TOYOBO, Japan). Quantitative PCR (qPCR) was conducted on LightCycler 480 (Roche) using SYBR mix (Roche) with the following procedure: 95 °C for 5 min, followed by 45 cycles at 95 °C for 10 s, 58 °C for 10 s and 72 °C for 20 s. The relative expression level was calculated by the 2^-ΔΔCT^ method [55]. Primers used in this section are listed in Table S2.

### Subcellular localization

The coding sequence of *PpSnRK1βγ* without stop codon was amplified from ‘JH’ using PCR primers listed in Table S2 and in-frame fused to the N-terminal of the GFP in the plant binary expression vector *pBWA(V)HS-Glosgfp* to generate the *35S:PpSnRK1βγ-GFP* construct. One step cloning kit (Novoprotein, China) was used to form recombination vector. The *35S:GFP* construct in the original vector was used as a control and *35S:NLS-mkate* was used as a nucleus localization marker. *Agrobacterium tumefaciens* strain GV3101 was transformed with these three vectors separately and cultivated in LB medium containing 50 μg/mL kanamycin under 28 °C. After cultivation, the Agrobacterium cells were re-suspended with infiltration buffer containing 10 mM MgCl_2_, 10 mM MES, and 200 μM acetosyringone to OD600 of 0.6–1.0, and placed at room temperature for 2 h. The cells containing *35S:PpSnRK1βγ-GFP* and *35S:GFP* were mixed with the same volume of cells containing *35S:NLS-mkate,* and injected into leaf tissues of tobacco (*N. benthamiana*) using a 1-mL syringe without needle. After infiltration, the tobacco plants were firstly placed in dark at room temperature for 12 h and then moved to conditions of 16 h light and 8 h dark for 48 h. The GFP and mkate (monomeric version of Katushka) fluorescence were observed using a confocal laser scanning microscope (TCS SP5, Leica, Germany).

### Promoter activity analysis

Based on the chromosome position of the 1.67-Mb inversion, promoter segments with different length were cloned using PCR. As shown in Fig. 2c, promoters of P1, P2, P3 and their recombination were used for luciferase activity analysis. The promoter regions of P2+P1, P3RC+P1 and P1, which could stand for the main part of promoter regions of two different alleles in flat and round peach were used for promoter activity analysis using *pGreen-II-0800-LUC* vector. The corresponding vectors were constructed using one step clone kit (NovoProtein, China) and named as *pGreen+P1*, *pGreen+P2+P1* and *pGreen+P3RC+P1*. The fragment of P2+P1 was the promoter region of *PpSnRK1βγ* in round peach, while P3RC+P1 was promoter in flat peach. P1 was truncated promoter (Fig. 2a). The transient expression assay was conducted following the protocol described above, using the empty vector *pGreen* as a negative control. Luciferase activities were evaluated using Tanon-5200Multi machine (Tanon, China). Primers used in this experiment are listed in Table S2.

### Sugar and starch contents quantification

For sugar extraction, 100 mg ground fresh sample and 10 mL 80% ethanol were added into a 15-mL tube and incubated in 80 °C water bath for 30 min, followed by centrifuging at 12000 x g for 15 min. The supernatant was transferred into a new tube for sugar analysis and the left item was used for starch quantification. For sucrose, fructose, glucose and starch quantification, the measurement kits were used (Bioengineering, China) according to the corresponding protocols.

### RNA-seq and data analysis

RNA was extracted from peach fruit in transient overexpression assay. Three biological replicates were conducted and the total RNA were mixed together to construct one library for experimental and control samples respectively. Total RNA of 20 μg was sent to ANBOROAD (Beijing, China) company for library construction and sequencing. The Illumina HiSeq™ X Ten platform in paired-end mode was used for sequencing. The raw reads were generated and the low quality reads were filtered to obtain clean reads using FASTX toolkit (http://hannonlab.cshl.edu/fastx_toolkit/). The clean reads were mapped to reference peach genome (release version 2.0_a2.1) [56] using tophat and the FPKM (fragments per kilobase of exon per million reads mapped) and differentially expressed genes (DEGs) were calculated using cufflink [57].

### Phylogenetic and gene structure analysis

The SnRK family genes in *Arabidopsis* were searched and download from the National Center for Biotechnology Information (NCBI), including 3 SnRK1αs, 3 SnRK1βs, 2 SnRK1γs (including 1 SnRK1βγ), 10 SnRK2s, and 25 SnRK3s. The amino acid sequences of these SnRK genes were downloaded. These 40 genes were used as queries in identifying SnRK family genes in peach genome. The local blast was performed using blast (Version 2.2.26) [43] with the following parameters: blastall -p blastp -m 8 -d -o. The blast results were further managed by keeping E-value equal to 0.0 or identity greater than 70%. The phylogenetic tree was constructed with Mega7 [58] using neighbor-joining method with bootstrap of 1000 and displayed with ITOL (https://itol.embl.de/). The gene structure was displayed using GSDS (http://gsds.cbi.pku.edu.cn/index.php).

### Gene transformation in tomato

The full-length coding sequence of *PpSnRK1βγ* was amplified from ‘JH’. Then, the overexpression vector was constructed using the *pBI121* vector driven by the cauliflower mosaic virus (CaMV) 35S promoter using a one-step construction kit (C112, Vazyme, China). Following protocol described in Sun et al. [59], Micro-Tom tomato transformation was performed using *Agrobacterium tumefaciens* GV3101. After transformation, transgenic lines were obtained and gene expression levels were analyzed. The three higher expression lines (OE1, OE3, OE9) and wild type (WT) were further used to determine the sugar and starch content in fruit at maturation stage. Primers used in this experiment are listed in Table S2.

### Transient overexpression in peach fruit

To further understand the function of *PpSnRK1βγ*, transient expression was carried out in peach fruits. The over-expression vector was the same as it used in tomato transformation. The peach cultivar used for transient expression was ‘Hakuho’ (HK), due to its white flesh and hesitation in browning during exposing in air. The peach fruits were collected at 50 DAFB and cut into 1 cm thick cubes and then infiltrated by submerging into *Agrobacterium tumefaciens* suspension under a vacuum of -70 kPa for 30 min as escribed by Liu et al. [60]. Then the fruit sections were cultivated on MS medium for 2 days. After that the sections were collected and stored under − 80 °C for gene expression and sugar/starch contents analysis. Three replicates were performed in this section.

## Supplementary Information

**Additional file 1: Figure S1.** Phenotype of aborting flat peach. The black arrow shows the aborting seed. **Figure S2.** Phylogenetic and gene structure analysis of SnRK gene family in peach and *Arabidopsis*. The referred SnRK genes in *Arabidopsis* were download from NCBI and used as queries to search for homologous genes in peach. The phylogenetic tree was constructed using neighbor-joining method with bootstrap of 1000. **Figure S3.** Flat peach cultivar and its bud mutation. **a** Relative gene expression of *PpSnRK1βγ* in flat peach ‘Zhongpantao 15’ (‘ZPT15’) and its bud mutation ‘ZPT15-Mut’ (round peach). **b** The bud mutation identified on the tree ‘ZPT15’. ‘ZPT15’ is flat peach and its bud mutation is round. **Figure S4.** Sugar and starch contents determination during peach fruit development. DAFB indicates days after full bloom. Fruit maturation stage is 70 DAFB. ‘XJ2’, aborting flat peach; ‘ZH’, viable flat peach; ‘JH’, round peach. **Figure S5.** The relationship between higher SSC and higher expression of *PpSnRK1βγ* in peach. **a** SSC in 28 round and 12 flat peach cultivars. **b** Relative gene expression of *PpSnRK1βγ* in 28 round and 12 flat peach cultivars. The SSC content and gene expression were determined at fruit maturation stage. ** indicates *P* < 0.01. **Figure S6.** Relative expression of *PpSnRK1βγ* in ‘HY’ (round peach) and ‘ZPT11’ (flat peach). **Table S1.** DEGs identified in transient expression assay. **Table S2.** Primers used in this study.

## Data Availability

Raw data generated in this study were deposited in the NCBI Short Read Archive (SRA) under the accession PRJNA633964. The data under accession PRJNA401307 generated in our previous study was also used. All other relevant data contained within the paper are available in the paper and Supplementary Files.

## Abbreviations

SnRK1: Sucrose non-fermenting 1 related kinase
DAFB: Days after full bloom
OE: Overexpression
DEGs: Differentially expressed genes
SSC: Soluble solid content
JH: Zhongnong Jin Hui
ZH: Zaohuang Pan Tao
XJ2: Xinjiang Pan Tao 2#
ZPT15: Zhongpantao 15#
ZPT15-Mut: Zhongpantao 15# mutant
T6P: Trehalose-6-phosphate phosphatase
VIN: Vacuolar invertase
CWIN: Cell wall bound invertase
INH: Invertase inhibitor

## References

[1] Shulaev V, Korban SS, Sosinski B, Abbott AG, Aldwinckle HS, Folta KM (2008). Multiple models for Rosaceae genomics. Plant Physiol.

[2] Faust M, Timon B (1995). Origin and dissemination of peach. Hortic Rev.

[3] Monet R, Bastard T, Gibault B (1985). Etude génétique et amélioration des pêches plates.

[4] Wang L, Shu H, Chen X (2008). Correlative analysis between peach fruit types and quality, yield. Acta Horticulturae Sinica.

[5] Dirlewanger E, Pronier V, Parvery C, Rothan C, Guye A, Monet R (1998). Genetic linkage map of peach [*Prunus persica* (L.) Batsch] using morphological and molecular markers. Theor Appl Genet.

[6] Dirlewanger E, Cosson P, Boudehri K, Renaud C, Capdeville G, Tauzin Y (2006). Development of a second-generation genetic linkage map for peach [*Prunus persica* (L.) Batsch] and characterization of morphological traits affecting flower and fruit. Tree Genet Genomes.

[7] Guo J, Cao K, Li Y, Yao JL, Deng C, Wang Q (2017). Comparative Transcriptome and microscopy analyses provide insights into flat shape formation in peach (*Prunus persica*). Front Plant Sci.

[8] Guo J, Cao K, Deng C, Li Y, Zhu G, Fang W (2020). An integrated peach genome structural variation map uncovers genes associated with fruit traits. Genome Biol.

[9] Lesley J (1940). A genetic study of saucer fruit shape and other characters in the peach.

[10] Cao K, Zhou Z, Wang Q, Guo J, Zhao P, Zhu G (2016). Genome-wide association study of 12 agronomic traits in peach. Nat Commun.

[11] Picañol R, Eduardo I, Aranzana MJ, Howad W, Batlle I, Iglesias I (2012). Combining linkage and association mapping to search for markers linked to the flat fruit character in peach. Euphytica.

[12] Zhou H, Ma R, Gao L, Zhang J (2020). A 1.7-Mb chromosomal inversion downstream of a *PpOFP1* gene is responsible for flat fruit shape in peach. Plant Biotechnol J.

[13] Boyer JS, McLaughlin JE (2007). Functional reversion to identify controlling genes in multigenic responses: analysis of floral abortion. J Exp Bot.

[14] Patrick JW, Stoddard FL (2010). Physiology of flowering and grain filling in faba bean. Field Crop Res.

[15] Ruan YL (2012). Signaling role of sucrose metabolism in development. Mol Plant.

[16] Weber H, Borisjuk L, Wobus U (2005). Molecular physiology of legume seed development. Annu Rev Plant Biol.

[17] Ruan YL, Patrick JW, Bouzayen M, Osorio S, Fernie AR (2012). Molecular regulation of seed and fruit set. Trends Plant Sci.

[18] Barnabas B, Jager K, Feher A (2008). The effect of drought and heat stress on reproductive processes in cereals. Plant Cell Environ.

[19] Liu YH, Offler CE, Ruan YL (2013). Regulation of fruit and seed response to heat and drought by sugars as nutrients and signals. Front Plant Sci.

[20] Zanor MI, Osorio S, Nunes-Nesi A, Carrari F, Lohse M, Usadel B (2009). RNA interference of *LIN5* in tomato confirms its role in controlling brix content, uncovers the influence of sugars on the levels of fruit hormones, and demonstrates the importance of sucrose cleavage for normal fruit development and fertility. Plant Physiol.

[21] Xu SM, Brill E, Llewellyn DJ, Furbank RT, Ruan YL (2012). Overexpression of a potato sucrose synthase gene in cotton accelerates leaf expansion, reduces seed abortion, and enhances fiber production. Mol Plant.

[22] Polge C, Thomas M (2007). SNF1/AMPK/SnRK1 kinases, global regulators at the heart of energy control?. Trends Plant Sci.

[23] Hardie DG (2007). AMP-activated/SNF1 protein kinases: conserved guardians of cellular energy. Nat Rev Mol Cell Biol.

[24] HEDBACKER K, CARLSON M (2008). SNF1/AMPK pathways in yeast. Front Biosci.

[25] Coello P, Hey SJ, Halford NG (2011). The sucrose non-fermenting-1-related (SnRK) family of protein kinases: potential for manipulation to improve stress tolerance and increase yield. J Exp Bot.

[26] Hrabak EM, Chan CW, Gribskov M, Harper JF, Choi JH, Halford N (2003). The Arabidopsis CDPK-SnRK superfamily of protein kinases. Plant Physiol.

[27] Fujii H (2009). Zhu J-k: Arabidopsis mutant deficient in 3 abscisic acid-activated protein kinases reveals critical roles in growth, reproduction, and stress. Proc Natl Acad Sci.

[28] Umezawa T, Nakashima K, Miyakawa T, Kuromori T, Tanokura M, Shinozaki K (2010). Molecular basis of the core regulatory network in ABA responses: sensing, signaling and transport. Plant Cell Physiol.

[29] Bai J, Mao J, Yang H, Khan A, Fan A, Liu S (2017). Sucrose non-ferment 1 related protein kinase 2 (SnRK2) genes could mediate the stress responses in potato (*Solanum tuberosum* L.). BMC Genet.

[30] Zhong R, Wang Y, Gai R, Xi D, Mao C, Ming F (2020). Rice SnRK protein kinase OsSAPK8 acts as a positive regulator in abiotic stress responses. Plant Sci.

[31] Lu K, Zhang YD, Zhao CF, Zhou LH, Zhao QY, Chen T (2020). The Arabidopsis kinase-associated protein phosphatase KAPP, interacting with protein kinases SnRK2.2/2.3/2.6, negatively regulates abscisic acid signaling. Plant Mol Biol.

[32] Emanuelle S, Doblin MS, Stapleton DI, Bacic A, Gooley PR (2016). Molecular insights into the enigmatic metabolic regulator, SnRK1. Trends Plant Sci.

[33] Wang WR, Liang JH, Wang GF, Sun MX, Peng FT, Xiao YS (2020). Overexpression of *PpSnRK1alpha* in tomato enhanced salt tolerance by regulating ABA signaling pathway and reactive oxygen metabolism. BMC Plant Biol.

[34] Yu W, Peng F, Xiao Y, Wang G, Luo J (2018). Overexpression of *PpSnRK1alpha* in tomato promotes fruit ripening by enhancing RIPENING INHIBITOR regulation pathway. Front Plant Sci.

[35] Emanuelle S, Hossain MI, Moller IE, Pedersen HL, van de Meene AM, Doblin MS (2015). SnRK1 from *Arabidopsis thaliana* is an atypical AMPK. Plant J.

[36] Bitrian M, Roodbarkelari F, Horvath M, Koncz C (2011). BAC-recombineering for studying plant gene regulation: developmental control and cellular localization of SnRK1 kinase subunits. Plant J.

[37] Tsai AY, Gazzarrini S (2012). *AKIN10* and *FUSCA3* interact to control lateral organ development and phase transitions in Arabidopsis. Plant J.

[38] Baena-González E, FRolland F, Thevelein JM, Sheen J (2007). A central integrator of transcription networks in plant stress and energy signalling. Nature.

[39] Ramon M, Ruelens P, Li Y, Sheen J, Geuten K, Rolland F (2013). The hybrid four-CBS-domain KINbetagamma subunit functions as the canonical gamma subunit of the plant energy sensor SnRK1. Plant J.

[40] Radchuk R, Radchuk V, Weschke W, Borisjuk L, Weber H (2006). Repressing the expression of the SUCROSE NONFERMENTING-1-RELATED PROTEIN KINASE gene in pea embryo causes pleiotropic defects of maturation similar to an abscisic acid-insensitive phenotype. Plant Physiol.

[41] Radchuk R, Emery RJ, Weier D, Vigeolas H, Geigenberger P, Lunn JE (2010). Sucrose non-fermenting kinase 1 (SnRK1) coordinates metabolic and hormonal signals during pea cotyledon growth and differentiation. Plant J.

[42] Gao XQ, Liu CZ, Li DD, Zhao TT, Li F, Jia XN (2016). The Arabidopsis KINbetagamma subunit of the SnRK1 complex regulates pollen hydration on the stigma by mediating the level of reactive oxygen species in pollen. PLoS Genet.

[43] Camacho C, Coulouris G, Avagyan V, Ma N, Papadopoulos J, Bealer K (2009). BLAST+: architecture and applications. BMC Bioinformatics.

[44] Lohse M, Nagel A, Herter T, May P, Schroda M, Zrenner R (2014). Mercator: a fast and simple web server for genome scale functional annotation of plant sequence data. Plant Cell Environ.

[45] Kleinow T, Bhalerao R (2000). Functional identification of an Arabidopsis Snf4 ortholog by screening for heterologous multicopy suppressors of snf4 deficiency in yeast. Plant J.

[46] Ruan YL, Jin Y, Yang YJ, Li GJ, Boyer JS (2010). Sugar input, metabolism, and signaling mediated by invertase: roles in development, yield potential, and response to drought and heat. Mol Plant.

[47] Reynolds M, Foulkes MJ, Slafer GA, Berry P, Parry MA, Snape JW (2009). Raising yield potential in wheat. J Exp Bot.

[48] Wubs AM, Heuvelink E, Marcelis LFM, Hemerik L (2011). Quantifying abortion rates of reproductive organs and effects of contributing factors using time-to-event analysis. Funct Plant Biol.

[49] McLaughlin JE, Boyer JS (2004). Glucose localization in maize ovaries when kernel number decreases at low water potential and sucrose is fed to the stems. Ann Bot.

[50] Punkkinen M, Denessiouk K, Fujii H (2019). Arabidopsis KIN gamma subunit 1 has a potential to regulate activity of sucrose nonfermenting 1-related protein kinase 2s (SnRK2s) in vitro. Biol Plant.

[51] Salazar JA, Ruiz D, Campoy JA, Sánchez-Pérez R, Crisosto CH, Martínez-García PJ (2013). Quantitative trait loci (QTL) and Mendelian trait loci (MTL) analysis in Prunus: a breeding perspective and beyond. Plant Mol Biol Report.

[52] Cirilli M, Bassi D, Ciacciulli A (2016). Sugars in peach fruit: a breeding perspective. Hortic Res.

[53] Sato R, Maeshima M (2019). The ER-localized aquaporin SIP2;1 is involved in pollen germination and pollen tube elongation in *Arabidopsis thaliana*. Plant Mol Biol.

[54] Guo WW, Liang WJ, Xie KD, Xia QM, Fu J, Guo DY (2016). Exploitation of polyploids from 39 citrus seedling populations. Acta Hortic.

[55] Livak KJ, Schmittgen TD (2001). Analysis of relative gene expression data using real-time quantitative PCR and the 2(−Delta Delta C(T)) method. Methods.

[56] Verde I, Jenkins J, Dondini L, Micali S, Pagliarani G, Vendramin E (2017). The peach v2.0 release: high-resolution linkage mapping and deep resequencing improve chromosome-scale assembly and contiguity. BMC Genomics.

[57] Trapnell C, Roberts A, Goff L, Pertea G, Kim D, Kelley DR (2012). Differential gene and transcript expression analysis of RNA-seq experiments with TopHat and cufflinks. Nat Protoc.

[58] Kumar S, Stecher G, Tamura K (2016). MEGA7: molecular evolutionary genetics analysis version 7.0 for bigger datasets. Mol Biol Evol.

[59] Sun HJ, Uchii S, Watanabe S, Ezura H (2006). A highly efficient transformation protocol for micro-tom, a model cultivar for tomato functional genomics. Plant Cell Physiol.

[60] Liu H, Cao X, Liu X, Xin R, Wang J, Gao J (2017). UV-B irradiation differentially regulates terpene synthases and terpene content of peach. Plant Cell Environ.

