# Supplementary material for: Reduced expression of a subunit gene of sucrose non-fermenting 1 related kinase, PpSnRK1βγ, confers flat fruit abortion in peach by regulating sugar and starch metabolism
Source: BMC Plant Biol. 2021 Feb 10;21:88. doi: 10.1186/s12870-021-02850-9 (PMC7877075; doi:10.1186/s12870-021-02850-9)
Supplement: Supplementary file 1 — Additional file 1: Figure S1. Phenotype of aborting flat peach. The black arrow shows the aborting seed. Figure S2. Phylogenetic and gene structure analysis of SnRK gene family in peach and Arabidopsis. The referred SnRK genes in Arabidopsis were download from NCBI and used as queries to search for homologous genes in peach. The phylogenetic tree was constructed using neighbor-joining method with bootstrap of 1000. Figure S3. Flat peach cultivar and its bud mutation. a Relative gene expression of PpSnRK1βγ in flat peach ‘Zhongpantao 15’ (‘ZPT15’) and its bud mutation ‘ZPT15-Mut’ (round peach). b The bud mutation identified on the tree ‘ZPT15’. ‘ZPT15’ is flat peach and its bud mutation is round. Figure S4. Sugar and starch contents determination during peach fruit development. DAFB indicates days after full bloom. Fruit maturation stage is 70 DAFB. ‘XJ2’, aborting flat peach; ‘ZH’, viable flat peach; ‘JH’, round peach. Figure S5. The relationship between higher SSC and higher expression of PpSnRK1βγ in peach. a SSC in 28 round and 12 flat peach cultivars. b Relative gene expression of PpSnRK1βγ in 28 round and 12 flat peach cultivars. The SSC content and gene expression were determined at fruit maturation stage. ** indicates P < 0.01. Figure S6. Relative expression of PpSnRK1βγ in ‘HY’ (round peach) and ‘ZPT11’ (flat peach). Table S1. DEGs identified in transient expression assay. Table S2. Primers used in this study. [file 12870_2021_2850_MOESM1_ESM.pdf]

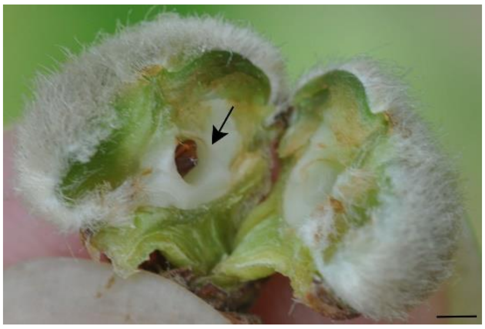

Supplementary Figure 1. Phenotype of aborting flat peach. The black arrow shows the aborting seed.

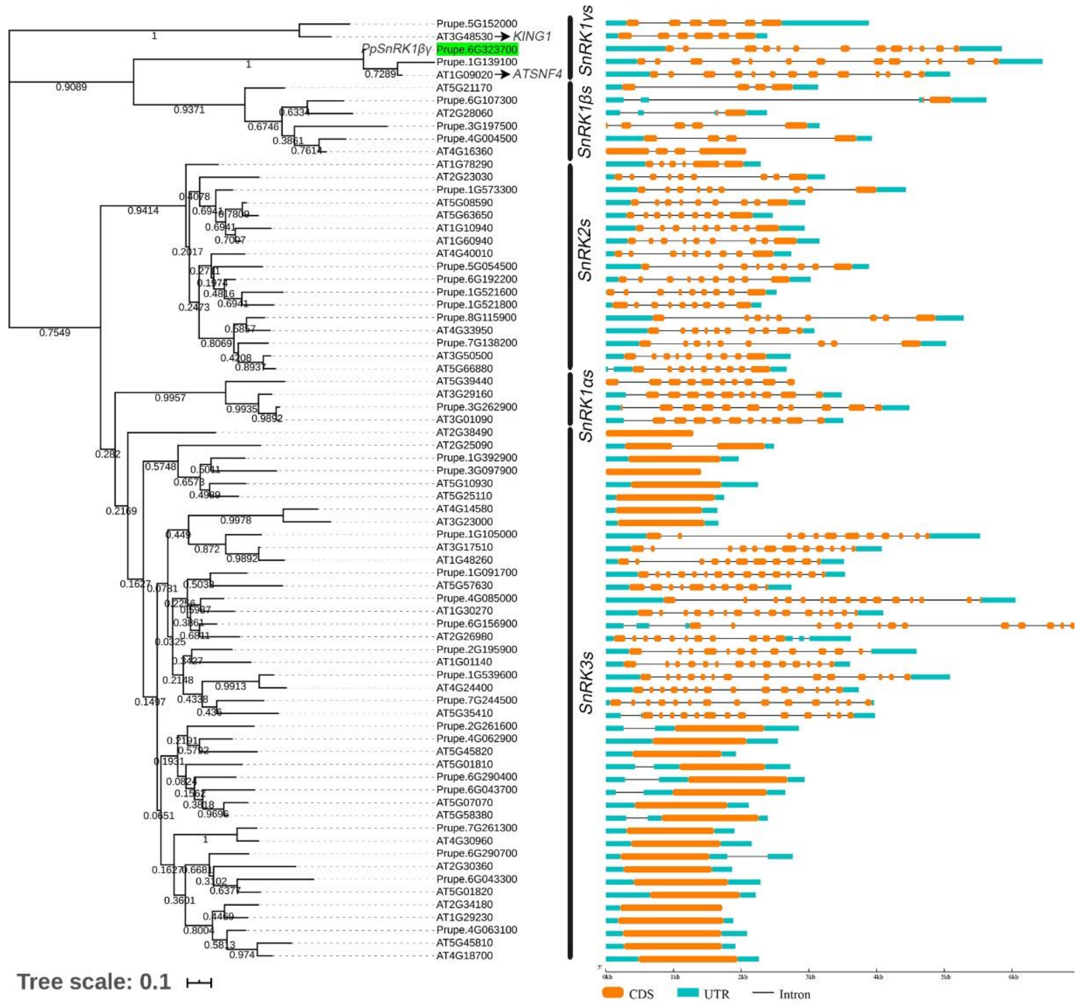

Supplementary Figure 2. Phylogenetic and gene structure analysis of SnRKs gene family in peach and *Arabidopsis*. The referred SnRKs in *Arabidopsis* were download from NCBI and used as queries to search for homologous genes in peach. The phylogenetic tree was constructed using neighbor-joining method with bootstrap of 1000.

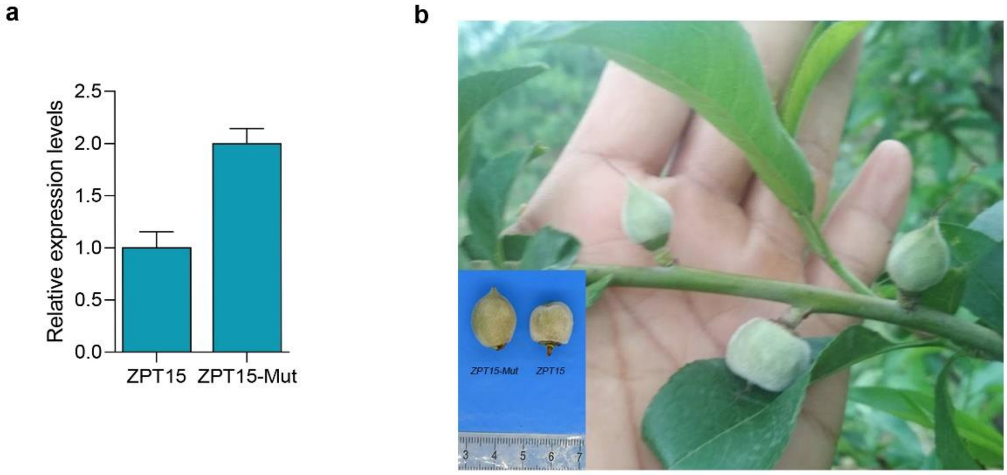

Supplementary Figure 3. Flat peach cultivar and its bud mutation. a Relative gene expression of *PpSnRK1βγ* in flat peach ‘Zhongpantao 15’(‘ZPT15’) and its bud mutation ‘ZPT15-Mut’ (round peach). **b** The bud mutation identified on the tree ‘ZPT15’. The cultivar ‘ZPT15’ is flat peach and its bud mutation is round.

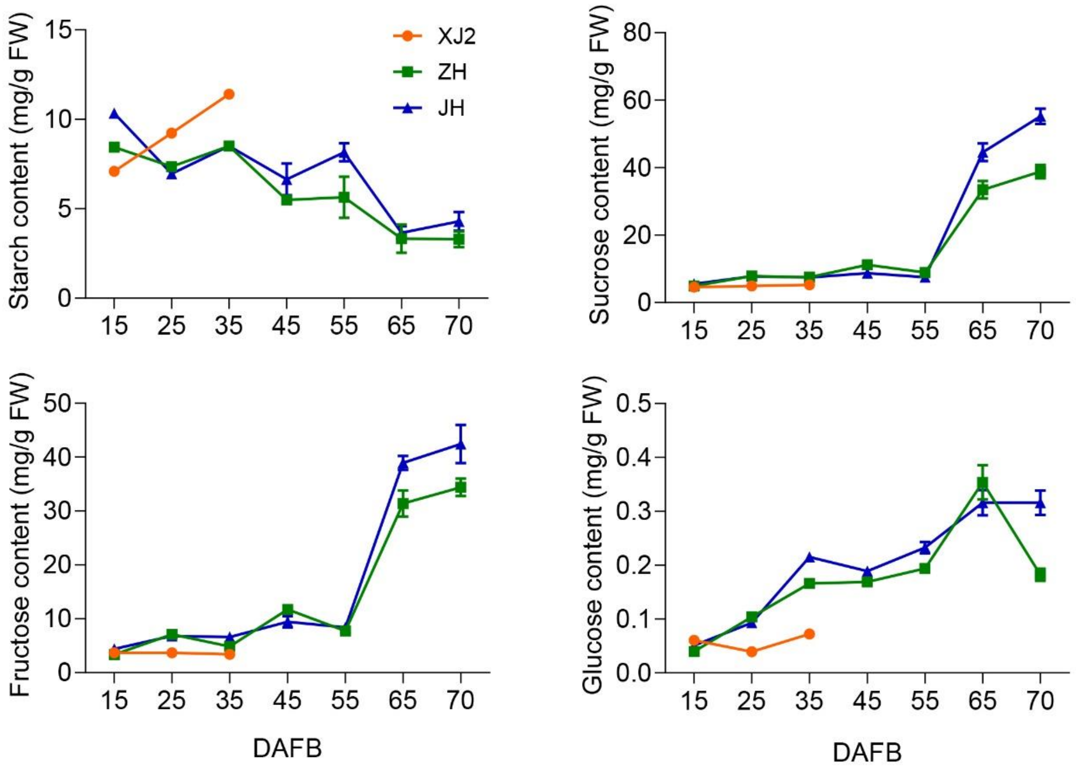

Supplementary Figure 4. Sugar and starch contents determination during peach fruit development. DAFB indicates days after full bloom. Fruit maturation stage is at 70 DAFB. 'XJ2', aborting flat peach; 'ZH', viable flat peach; 'JH', round peach.

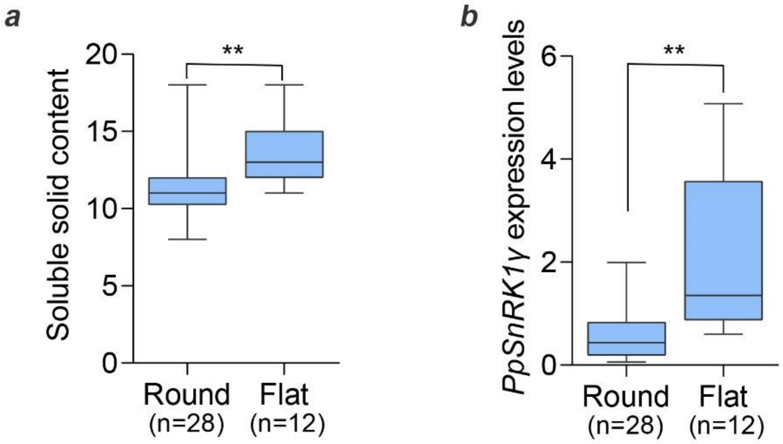

Supplementary Figure 5. The relationship between higher SSC and higher expression of *PpSnRK1βγ* in peach. a SSC in 28 round and 12 flat peach cultivars. **b** Relative gene expression of *PpSnRK1βγ* in 28 round and 12 flat peach cultivars. The SSC content and gene expression were determined at fruit maturation stage. ** indicates P <0.01.

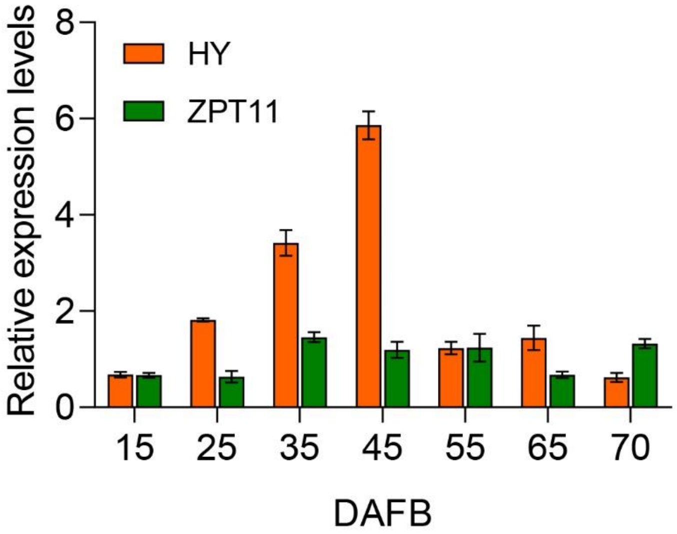

Supplementary Figure 6. Relative expression of *PpSnRK1βγ* in 'HY' (round peach) and 'ZPT11' (flat peach).

Supplementary Table 1. DEGs identified in transient expression assay.

GeneID	FS1_FPKM	CK_FPKM	Up/Down
Prupe.1G006800_v2.0.a1	2.240370261	3.751714631	down
Prupe.1G011900_v2.0.a1	5.49795914	14.9762739	down
Prupe.1G021800_v2.0.a1	65.29715066	21.19543887	up
Prupe.1G023600_v2.0.a1	28.33499691	56.93278148	down
Prupe.1G032000_v2.0.a1	6.234233683	3.120598832	up
Prupe.1G032400_v2.0.a1	58.9552395	15.573444	up
Prupe.1G032700_v2.0.a1	9.005757136	17.6023095	down
Prupe.1G036500_v2.0.a1	5.481366684	31.31322778	down
Prupe.1G038100_v2.0.a1	3.273458822	1.862781416	up
Prupe.1G043400_v2.0.a1	0.057636846	0.634713521	down
Prupe.1G051500_v2.0.a1	0.580987488	0.830909261	down
Prupe.1G066500_v2.0.a1	1.512736111	0.409304303	up
Prupe.1G073400_v2.0.a1	0.994287919	0.497699296	up
Prupe.1G080700_v2.0.a1	161.6090714	318.9469849	down
Prupe.1G081600_v2.0.a1	1.33350813	3.091573694	down
Prupe.1G083700_v2.0.a1	0.124886038	0.017860792	up
Prupe.1G085900_v2.0.a1	0.052755788	0.792220792	down
Prupe.1G088600_v2.0.a1	30.35935949	91.78232748	down
Prupe.1G088900_v2.0.a1	0.477814104	4.305130638	down
Prupe.1G093800_v2.0.a1	14.49243294	3.079660283	up
Prupe.1G095900_v2.0.a1	5.654008154	12.97062097	down
Prupe.1G098800_v2.0.a1	2.390399833	0.854667872	up
Prupe.1G098900_v2.0.a1	3.711812861	1.415603489	up
Prupe.1G104100_v2.0.a1	0.152073618	0.326236055	down
Prupe.1G114500_v2.0.a1	18.53945232	7.746183276	up
Prupe.1G121100_v2.0.a1	0.792851346	2.116632012	down
Prupe.1G126900_v2.0.a1	2.248765819	6.824185874	down
Prupe.1G127400_v2.0.a1	50.26783956	101.6871215	down
Prupe.1G129000_v2.0.a1	41.16593891	101.1517971	down
Prupe.1G137800_v2.0.a1	2.608334104	0.921616855	up
Prupe.1G140300_v2.0.a1	1.797761681	0.550949963	up
Prupe.1G141900_v2.0.a1	3.249135908	0.451772968	up
Prupe.1G166300_v2.0.a1	1.05596917	0.508244589	up
Prupe.1G169700_v2.0.a1	0.044724948	0.537298895	down
Prupe.1G184500_v2.0.a1	1.200767618	1.63486816	down
Prupe.1G194800_v2.0.a1	0.445446052	2.229718198	down
Prupe.1G210900_v2.0.a1	1.541028902	2.389750489	down
Prupe.1G242100_v2.0.a1	1.170430376	3.244812399	down
Prupe.1G257200_v2.0.a1	0.572230588	1.326645855	down
Prupe.1G270600_v2.0.a1	0.392435598	1.375058896	down
Prupe.1G275300_v2.0.a1	3.049500665	1.575694	up

Prupe.1G290400_v2.0.a1	1.552799892	0.355322161	up
Prupe.1G301000_v2.0.a1	2.678848774	0.383120171	up
Prupe.1G302900_v2.0.a1	0.743909905	2.978963572	down
Prupe.1G303000_v2.0.a1	0.267483343	1.87447496	down
Prupe.1G308600_v2.0.a1	14.98140538	34.55372685	down
Prupe.1G309300_v2.0.a1	8.155946803	2.135172407	up
Prupe.1G315100_v2.0.a1	0.194948154	0.520442459	down
Prupe.1G319000_v2.0.a1	10.48261366	20.94828385	down
Prupe.1G319100_v2.0.a1	0.753779824	3.186181091	down
Prupe.1G322400_v2.0.a1	0.567243239	1.597153728	down
Prupe.1G324300_v2.0.a1	1.218146061	0.685972573	up
Prupe.1G339600_v2.0.a1	2.484633341	0.956695697	up
Prupe.1G339700_v2.0.a1	0.816651095	0	up
Prupe.1G341900_v2.0.a1	1.143081073	2.574805405	down
Prupe.1G343000_v2.0.a1	0.306862836	0.71681311	down
Prupe.1G343400_v2.0.a1	10.41442725	23.20400374	down
Prupe.1G356000_v2.0.a1	0.027196115	2.368705063	down
Prupe.1G360900_v2.0.a1	0.765113517	0.05892063	up
Prupe.1G369800_v2.0.a1	110.640141	377.2076434	down
Prupe.1G370500_v2.0.a1	1.266138921	3.290763291	down
Prupe.1G373700_v2.0.a1	0.444333981	0.122711814	up
Prupe.1G392300_v2.0.a1	1.907997168	0.65282873	up
Prupe.1G399700_v2.0.a1	1.707501088	2.289666379	down
Prupe.1G399900_v2.0.a1	0.531619927	1.383755817	down
Prupe.1G405200_v2.0.a1	0.27402351	1.097318438	down
Prupe.1G407100_v2.0.a1	1.908654706	0.579026296	up
Prupe.1G429900_v2.0.a1	1.439490592	4.261534378	down
Prupe.1G442500_v2.0.a1	0.646447066	2.109292152	down
Prupe.1G449100_v2.0.a1	0.220843089	0.859793601	down
Prupe.1G454200_v2.0.a1	1.144773273	2.363732349	down
Prupe.1G458400_v2.0.a1	0.305918642	0.986838971	down
Prupe.1G462000_v2.0.a1	0.894633102	0.431230442	up
Prupe.1G464100_v2.0.a1	0.408142688	2.088392874	down
Prupe.1G473900_v2.0.a1	1.044935876	3.703205071	down
Prupe.1G474000_v2.0.a1	0.384580055	0.914397924	down
Prupe.1G481200_v2.0.a1	0.386102088	0.795804028	down
Prupe.1G492800_v2.0.a1	0.286713265	1.234244218	down
Prupe.1G497100_v2.0.a1	34.5696128	13.15789634	up
Prupe.1G498800_v2.0.a1	3.179010672	0.63651236	up
Prupe.1G498900_v2.0.a1	1.781784207	0.20985583	up
Prupe.1G510500_v2.0.a1	2.420326592	0.865367939	up
Prupe.1G513200_v2.0.a1	0.558800806	0.215163468	up
Prupe.1G520800_v2.0.a1	15.47120653	31.99770879	down
Prupe.1G520900_v2.0.a1	93.75686207	202.5557189	down

Prupe.1G521700_v2.0.a1	23.46702495	46.98647782	down
Prupe.1G528800_v2.0.a1	0.656001312	0.341501733	up
Prupe.1G529400_v2.0.a1	37.11486576	189.485592	down
Prupe.1G538900_v2.0.a1	1.024773848	0.153887787	up
Prupe.1G540400_v2.0.a1	1.39002768	0.183102686	up
Prupe.1G552700_v2.0.a1	17.26773782	38.6542215	down
Prupe.1G552800_v2.0.a1	1.666909231	0.83438563	up
Prupe.1G565100_v2.0.a1	0.306522255	1.59569622	down
Prupe.1G572200_v2.0.a1	1.617081466	0.599588087	up
Prupe.1G581000_v2.0.a1	1.311838623	0.783374327	up
Prupe.2G013300_v2.0.a1	3.777302619	7.501051982	down
Prupe.2G014800_v2.0.a1	0.430142133	0.65859931	down
Prupe.2G031400_v2.0.a1	4.366537632	11.1095204	down
Prupe.2G033700_v2.0.a1	0.374823205	1.262177309	down
Prupe.2G043900_v2.0.a1	1.699104961	0.634272288	up
Prupe.2G045000_v2.0.a1	3.105072875	0.700516374	up
Prupe.2G045300_v2.0.a1	3.628986579	1.763093114	up
Prupe.2G058000_v2.0.a1	2.668205084	4.856701119	down
Prupe.2G063000_v2.0.a1	3.256902766	7.137940902	down
Prupe.2G085000_v2.0.a1	40.1539162	13.60500835	up
Prupe.2G086100_v2.0.a1	2.570064025	5.441124682	down
Prupe.2G088100_v2.0.a1	0.125477761	0.452224536	down
Prupe.2G090500_v2.0.a1	1.809432583	0.699157614	up
Prupe.2G093800_v2.0.a1	1.766856845	0.505380149	up
Prupe.2G095000_v2.0.a1	0.128828712	1.289726211	down
Prupe.2G098600_v2.0.a1	4.110122136	10.27198229	down
Prupe.2G109000_v2.0.a1	7.261464487	15.09039935	down
Prupe.2G110400_v2.0.a1	1.12951116	3.990963126	down
Prupe.2G121000_v2.0.a1	1.353676517	2.710377296	down
Prupe.2G123600_v2.0.a1	0.968301936	0.337176898	up
Prupe.2G136300_v2.0.a1	0.2601349	0.028936165	up
Prupe.2G137300_v2.0.a1	1.773204187	0.133138872	up
Prupe.2G141000_v2.0.a1	111.6529354	237.7285892	down
Prupe.2G145500_v2.0.a1	2.114270256	3.174948018	down
Prupe.2G156900_v2.0.a1	0.473136531	0.28419903	up
Prupe.2G164900_v2.0.a1	1.744040577	3.956101129	down
Prupe.2G180700_v2.0.a1	0.265554377	2.392659143	down
Prupe.2G182500_v2.0.a1	3.103107328	1.242629468	up
Prupe.2G184700_v2.0.a1	18.151653	8.185859782	up
Prupe.2G185900_v2.0.a1	0.618690472	0.17205044	up
Prupe.2G189400_v2.0.a1	15.85241732	7.861995794	up
Prupe.2G193900_v2.0.a1	0.990894857	0.651886858	up
Prupe.2G195800_v2.0.a1	1.97476399	7.791587292	down
Prupe.2G207600_v2.0.a1	5.163370046	1.882587229	up

Prupe.2G213600_v2.0.a1	159.8002692	73.84527506	up
Prupe.2G238800_v2.0.a1	19.14292347	7.985127987	up
Prupe.2G248400_v2.0.a1	0.214031095	0.550980461	down
Prupe.2G248900_v2.0.a1	6.712233922	2.440126093	up
Prupe.2G261600_v2.0.a1	0.418115896	0.793104545	down
Prupe.2G269300_v2.0.a1	0.470421381	0.824157017	down
Prupe.2G269800_v2.0.a1	101.0446748	255.8496513	down
Prupe.2G269900_v2.0.a1	0.087121941	0.828582977	down
Prupe.2G277800_v2.0.a1	0.306773136	0.74354356	down
Prupe.2G279700_v2.0.a1	49.13196121	16.41016279	up
Prupe.2G288800_v2.0.a1	26.40892495	69.78130804	down
Prupe.2G302500_v2.0.a1	0.222822857	0.570072282	down
Prupe.2G305200_v2.0.a1	6.540498853	15.97025597	down
Prupe.2G316500_v2.0.a1	2.761765522	1.311166247	up
Prupe.2G319600_v2.0.a1	0.570971748	0.994103574	down
Prupe.2G320200_v2.0.a1	1.389726475	3.086109506	down
Prupe.2G324700_v2.0.a1	0.852584726	0.121933873	up
Prupe.3G013700_v2.0.a1	0.890892104	0.288551767	up
Prupe.3G014800_v2.0.a1	3.8263567	1.791746735	up
Prupe.3G027700_v2.0.a1	0.614864309	0.068394572	up
Prupe.3G032700_v2.0.a1	5.014039893	11.61803971	down
Prupe.3G033300_v2.0.a1	0.398650998	0.133032104	up
Prupe.3G035700_v2.0.a1	12.52565437	5.751266308	up
Prupe.3G039300_v2.0.a1	0.797088927	0.591095792	up
Prupe.3G052000_v2.0.a1	3.75029414	0.857001651	up
Prupe.3G055200_v2.0.a1	0.81754391	2.404217883	down
Prupe.3G060600_v2.0.a1	1.448267426	0.201372947	up
Prupe.3G063600_v2.0.a1	0.292559907	0.029288671	up
Prupe.3G091300_v2.0.a1	35.3743133	69.04707315	down
Prupe.3G091600_v2.0.a1	0.542974455	1.556618281	down
Prupe.3G097600_v2.0.a1	2.451497828	1.112075817	up
Prupe.3G099800_v2.0.a1	0.481538604	0.028357442	up
Prupe.3G103100_v2.0.a1	2.75906056	8.719705015	down
Prupe.3G143900_v2.0.a1	161.0556245	368.9743514	down
Prupe.3G146100_v2.0.a1	0.164146539	0.684707916	down
Prupe.3G156800_v2.0.a1	0.565428642	0.672196555	down
Prupe.3G157200_v2.0.a1	254.4303669	120.3596365	up
Prupe.3G167900_v2.0.a1	0.307754879	0.120560344	up
Prupe.3G171500_v2.0.a1	1.458567325	4.253616254	down
Prupe.3G171600_v2.0.a1	1.406741989	12.1363478	down
Prupe.3G171700_v2.0.a1	0.73386152	3.134640145	down
Prupe.3G171800_v2.0.a1	0.388373276	1.641630023	down
Prupe.3G173200_v2.0.a1	0.698689802	1.670956772	down
Prupe.3G174700_v2.0.a1	14.53560801	6.462005782	up

Prupe.3G178000_v2.0.a1	3.656693025	6.936210478	down
Prupe.3G181200_v2.0.a1	2.354847819	0.867552019	up
Prupe.3G189300_v2.0.a1	2.832580022	6.573770575	down
Prupe.3G189700_v2.0.a1	21.63647312	9.730278036	up
Prupe.3G195700_v2.0.a1	0.937636443	0.455119435	up
Prupe.3G197100_v2.0.a1	0.927389363	1.931124639	down
Prupe.3G217600_v2.0.a1	2.852081434	9.826878515	down
Prupe.3G220200_v2.0.a1	0.113032695	0.037719653	up
Prupe.3G228800_v2.0.a1	0.632163782	1.147076772	down
Prupe.3G233900_v2.0.a1	3.184297275	1.072835585	up
Prupe.3G242500_v2.0.a1	0.198598869	0.944398395	down
Prupe.3G244700_v2.0.a1	13.47892388	3.868903521	up
Prupe.3G254800_v2.0.a1	0.309614969	0.061992165	up
Prupe.3G267200_v2.0.a1	1.447546066	0.915260877	up
Prupe.3G272200_v2.0.a1	3.729262997	1.479283105	up
Prupe.3G274100_v2.0.a1	0.459618344	0.216532594	up
Prupe.3G276100_v2.0.a1	0.247108759	0.11718227	up
Prupe.3G277200_v2.0.a1	0.314859261	0.067545209	up
Prupe.3G285600_v2.0.a1	0.472514455	0.146806225	up
Prupe.3G289900_v2.0.a1	0.139880495	1.085284806	down
Prupe.3G295700_v2.0.a1	0.746983913	0.41545463	up
Prupe.3G298500_v2.0.a1	10.73370342	4.213997475	up
Prupe.3G308000_v2.0.a1	0.077306243	0.309570392	down
Prupe.3G310500_v2.0.a1	13.70426188	5.0740242	up
Prupe.3G315500_v2.0.a1	42.4212197	17.69002517	up
Prupe.4G005100_v2.0.a1	0.135491358	0.949498968	down
Prupe.4G009800_v2.0.a1	65.8246957	29.37794102	up
Prupe.4G018400_v2.0.a1	0.100610766	1.41012415	down
Prupe.4G021100_v2.0.a1	4.277734639	15.58985033	down
Prupe.4G025200_v2.0.a1	9.255417121	4.016577685	up
Prupe.4G027900_v2.0.a1	8.523236929	16.28334624	down
Prupe.4G028700_v2.0.a1	0.099955321	0.480321488	down
Prupe.4G028800_v2.0.a1	4.886115117	10.18336597	down
Prupe.4G031800_v2.0.a1	0	0.179795196	down
Prupe.4G044100_v2.0.a1	43.76020884	94.22829544	down
Prupe.4G081200_v2.0.a1	0.579290094	1.391849262	down
Prupe.4G082500_v2.0.a1	10.28157273	20.51098381	down
Prupe.4G093100_v2.0.a1	0.08494473	1.21181455	down
Prupe.4G093500_v2.0.a1	0.552353104	1.682952518	down
Prupe.4G099400_v2.0.a1	14.06879446	43.99878474	down
Prupe.4G103400_v2.0.a1	0.556909638	0.354792923	up
Prupe.4G109500_v2.0.a1	0.010179748	0.560511541	down
Prupe.4G122300_v2.0.a1	0.330646156	0.087109344	up
Prupe.4G123100_v2.0.a1	2.894274473	6.52811939	down

Prupe.4G145000_v2.0.a1	0.87213648	0.194024601	up
Prupe.4G149500_v2.0.a1	3.465253791	0.919527021	up
Prupe.4G152800_v2.0.a1	28.80683413	12.27454074	up
Prupe.4G153000_v2.0.a1	10.29671627	3.865581851	up
Prupe.4G157400_v2.0.a1	0.45246316	1.254374548	down
Prupe.4G171600_v2.0.a1	0.06485555	0.302997322	down
Prupe.4G177600_v2.0.a1	38.41076185	15.88994578	up
Prupe.4G183100_v2.0.a1	0.987146084	0.49412439	up
Prupe.4G185300_v2.0.a1	6.539758171	2.310728273	up
Prupe.4G191500_v2.0.a1	4.821844689	12.50036673	down
Prupe.4G193000_v2.0.a1	0.795486376	0.204198712	up
Prupe.4G197000_v2.0.a1	0.295639485	0.591939461	down
Prupe.4G202900_v2.0.a1	0.878057593	1.758076862	down
Prupe.4G208300_v2.0.a1	0.374592118	1.090939804	down
Prupe.4G235800_v2.0.a1	23.07421017	1.494513819	up
Prupe.4G240700_v2.0.a1	27.72786134	7.369992728	up
Prupe.4G242100_v2.0.a1	3.677546778	1.443786196	up
Prupe.4G243900_v2.0.a1	1.496891882	0.786746096	up
Prupe.4G262900_v2.0.a1	40.28879408	84.13672242	down
Prupe.4G270100_v2.0.a1	1.459562291	3.744306298	down
Prupe.4G276500_v2.0.a1	0.111473886	0.35339496	down
Prupe.4G280000_v2.0.a1	16.63947582	72.92481928	down
Prupe.5G008000_v2.0.a1	9.454424086	3.105231736	up
Prupe.5G018200_v2.0.a1	1.763434216	4.641304245	down
Prupe.5G021800_v2.0.a1	0.826758032	0.433547487	up
Prupe.5G022600_v2.0.a1	2.079152275	9.153473527	down
Prupe.5G040300_v2.0.a1	0.81940447	0.223723579	up
Prupe.5G041300_v2.0.a1	14.57327389	6.122401723	up
Prupe.5G062700_v2.0.a1	5.418078508	22.64301582	down
Prupe.5G064300_v2.0.a1	0.971805272	0.394415202	up
Prupe.5G072800_v2.0.a1	158.6771883	455.254386	down
Prupe.5G075600_v2.0.a1	3.209453641	8.452133853	down
Prupe.5G077600_v2.0.a1	8.062123593	3.909889688	up
Prupe.5G078200_v2.0.a1	0.572080001	0.059246798	up
Prupe.5G084500_v2.0.a1	0.992136837	2.196599758	down
Prupe.5G096000_v2.0.a1	1.386660513	6.2469427	down
Prupe.5G096100_v2.0.a1	2.353073763	3.843514239	down
Prupe.5G104600_v2.0.a1	0.676948157	0.164847003	up
Prupe.5G105900_v2.0.a1	0.242127388	0.787793038	down
Prupe.5G107700_v2.0.a1	0.115918805	1.276531216	down
Prupe.5G108200_v2.0.a1	0.905496892	2.590023949	down
Prupe.5G108400_v2.0.a1	0.126252138	1.074341934	down
Prupe.5G110100_v2.0.a1	1.114613888	2.107733615	down
Prupe.5G110800_v2.0.a1	1.607915966	0.770848046	up

Prupe.5G111300_v2.0.a1	113.6243498	47.53919243	up
Prupe.5G115800_v2.0.a1	0.889902224	0.33792618	up
Prupe.5G120000_v2.0.a1	5.705055538	10.25461008	down
Prupe.5G123100_v2.0.a1	8.426815605	3.32726582	up
Prupe.5G131200_v2.0.a1	0.478227796	0.215442901	up
Prupe.5G140400_v2.0.a1	8.325682337	4.727303554	up
Prupe.5G148300_v2.0.a1	1.940999865	0.610709964	up
Prupe.5G153300_v2.0.a1	7.237730332	1.906793493	up
Prupe.5G165500_v2.0.a1	0.833925646	0.049109246	up
Prupe.5G185300_v2.0.a1	0.837986938	3.547445892	down
Prupe.5G194800_v2.0.a1	1.754297518	0.301072657	up
Prupe.5G195500_v2.0.a1	2.259928465	0.532341866	up
Prupe.5G202800_v2.0.a1	14.42735577	29.93140979	down
Prupe.5G219300_v2.0.a1	23.62190935	64.23865569	down
Prupe.5G219700_v2.0.a1	15.65642598	33.24132322	down
Prupe.5G226200_v2.0.a1	8.358854484	30.6610547	down
Prupe.5G228100_v2.0.a1	104.9206614	44.84614075	up
Prupe.5G237900_v2.0.a1	2.156839456	0.641026977	up
Prupe.5G239000_v2.0.a1	0.523557445	0.052414229	up
Prupe.5G240700_v2.0.a1	0.46986559	1.986093034	down
Prupe.6G005400_v2.0.a1	0.589234841	0.131087344	up
Prupe.6G013600_v2.0.a1	2.682080521	7.199118291	down
Prupe.6G020900_v2.0.a1	2.43396535	7.48690878	down
Prupe.6G032400_v2.0.a1	24.51201609	57.76860529	down
Prupe.6G068900_v2.0.a1	0.749184235	0.102275607	up
Prupe.6G077800_v2.0.a1	356.5685386	762.2957631	down
Prupe.6G083300_v2.0.a1	1.792626708	0.560821616	up
Prupe.6G085200_v2.0.a1	1.021157	2.354382554	down
Prupe.6G090800_v2.0.a1	3.081077154	0.897314585	up
Prupe.6G097800_v2.0.a1	14.24609659	36.49247465	down
Prupe.6G103800_v2.0.a1	0.901119868	1.63719248	down
Prupe.6G106900_v2.0.a1	3.377158926	6.548989337	down
Prupe.6G113300_v2.0.a1	3.660944994	15.04593098	down
Prupe.6G120400_v2.0.a1	2.044670402	1.111203823	up
Prupe.6G129600_v2.0.a1	1.369470507	0.457000093	up
Prupe.6G151500_v2.0.a1	1.580528461	0.58295042	up
Prupe.6G154000_v2.0.a1	0.524801049	1.116447972	down
Prupe.6G155500_v2.0.a1	11.48894457	25.87900129	down
Prupe.6G183500_v2.0.a1	0.112587261	0.383224294	down
Prupe.6G184400_v2.0.a1	0.028109573	0.281409727	down
Prupe.6G194200_v2.0.a1	0.173332564	1.272525365	down
Prupe.6G195100_v2.0.a1	46.43287052	20.59868748	up
Prupe.6G195800_v2.0.a1	4.908127379	12.81318307	down
Prupe.6G205900_v2.0.a1	0.826979071	0.206975713	up

Prupe.6G208400_v2.0.a1	0.723539462	1.664458534	down
Prupe.6G209900_v2.0.a1	84.1762227	157.5152888	down
Prupe.6G210200_v2.0.a1	4.276492711	2.712560247	up
Prupe.6G210900_v2.0.a1	2.429221595	7.602748779	down
Prupe.6G225300_v2.0.a1	4.309466334	12.71089018	down
Prupe.6G226900_v2.0.a1	60.53184705	23.74576841	up
Prupe.6G227000_v2.0.a1	26.10608458	7.25103341	up
Prupe.6G235800_v2.0.a1	2.965654251	1.298923084	up
Prupe.6G245600_v2.0.a1	0.345810139	1.730982141	down
Prupe.6G261400_v2.0.a1	3.175982519	1.321611978	up
Prupe.6G267100_v2.0.a1	0.805224598	0.356554881	up
Prupe.6G267800_v2.0.a1	1.78527448	2.383024973	down
Prupe.6G273500_v2.0.a1	0.47383232	1.490850795	down
Prupe.6G278400_v2.0.a1	72.82058537	17.87223846	up
Prupe.6G282800_v2.0.a1	4.362265802	9.908887102	down
Prupe.6G291400_v2.0.a1	0.340328468	1.669472307	down
Prupe.6G298800_v2.0.a1	1.881572353	4.501662006	down
Prupe.6G306500_v2.0.a1	0.490782241	4.109309402	down
Prupe.6G321200_v2.0.a1	12.97422814	25.74956983	down
Prupe.6G356300_v2.0.a1	2.247552706	9.136620857	down
Prupe.7G016400_v2.0.a1	1.855164365	0.8401785	up
Prupe.7G029800_v2.0.a1	0.583829108	0.279534524	up
Prupe.7G050600_v2.0.a1	0.477969155	2.392515362	down
Prupe.7G051200_v2.0.a1	5.48840525	7.956088272	down
Prupe.7G066100_v2.0.a1	2.5171031	8.547551146	down
Prupe.7G068200_v2.0.a1	10.79599249	51.77811059	down
Prupe.7G069000_v2.0.a1	0.424524162	0.129860616	up
Prupe.7G077300_v2.0.a1	1.465217982	3.001935222	down
Prupe.7G090800_v2.0.a1	1.193025725	4.492213685	down
Prupe.7G093400_v2.0.a1	9.979488859	20.63957075	down
Prupe.7G100900_v2.0.a1	4.884266722	13.33887594	down
Prupe.7G105400_v2.0.a1	0	0.680939842	down
Prupe.7G106800_v2.0.a1	0.070006731	1.401698639	down
Prupe.7G113300_v2.0.a1	23.2373522	66.89756542	down
Prupe.7G113800_v2.0.a1	0.045237764	0.769901058	down
Prupe.7G123400_v2.0.a1	9.174249728	34.33101962	down
Prupe.7G124000_v2.0.a1	6.326511327	17.00765105	down
Prupe.7G132700_v2.0.a1	0.498738695	0.062411977	up
Prupe.7G138500_v2.0.a1	1.509677087	3.589488264	down
Prupe.7G140400_v2.0.a1	0.232276326	1.023157484	down
Prupe.7G140700_v2.0.a1	1.606981291	0.598614466	up
Prupe.7G148000_v2.0.a1	0.987561037	2.522798296	down
Prupe.7G148300_v2.0.a1	0.366403386	0.94759942	down
Prupe.7G160000_v2.0.a1	2.294337135	1.062744793	up

Prupe.7G162900_v2.0.a1	1.941574679	0.797433241	up
Prupe.7G165900_v2.0.a1	2.095132465	0.381358699	up
Prupe.7G169400_v2.0.a1	0.30441996	0.982004487	down
Prupe.7G170700_v2.0.a1	7.696622321	3.537227742	up
Prupe.7G180800_v2.0.a1	1.27041214	0.525321607	up
Prupe.7G190300_v2.0.a1	0.151246743	0.635945913	down
Prupe.7G193500_v2.0.a1	1.134486479	0.567876881	up
Prupe.7G196900_v2.0.a1	0.339015496	1.115152354	down
Prupe.7G210100_v2.0.a1	0.973447768	0.3458028	up
Prupe.7G216300_v2.0.a1	3.160145576	22.78519577	down
Prupe.7G221500_v2.0.a1	10.04883358	3.064653639	up
Prupe.7G228300_v2.0.a1	0.211710657	1.377656472	down
Prupe.7G234200_v2.0.a1	1.462925187	3.404110932	down
Prupe.7G238400_v2.0.a1	0.692244099	1.761419159	down
Prupe.7G243500_v2.0.a1	10.3956602	5.404635192	up
Prupe.7G246200_v2.0.a1	178.2960956	383.9118628	down
Prupe.8G005400_v2.0.a1	0.180147778	0.681318494	down
Prupe.8G009700_v2.0.a1	0.396484956	0.942703647	down
Prupe.8G009800_v2.0.a1	0.313836991	1.047291881	down
Prupe.8G024400_v2.0.a1	0.080051175	0.320562384	down
Prupe.8G033100_v2.0.a1	1.869502815	2.83574417	down
Prupe.8G035600_v2.0.a1	0	0.881546557	down
Prupe.8G047100_v2.0.a1	7.69067024	13.38447715	down
Prupe.8G056300_v2.0.a1	1.3511573	3.192293216	down
Prupe.8G066000_v2.0.a1	4.193957335	11.70259852	down
Prupe.8G094600_v2.0.a1	2.133552763	1.364625816	up
Prupe.8G096000_v2.0.a1	0.50496416	1.438811124	down
Prupe.8G097600_v2.0.a1	0.049317241	0.263319101	down
Prupe.8G103800_v2.0.a1	0.910228186	0.511186198	up
Prupe.8G106100_v2.0.a1	0.159639626	1.171998313	down
Prupe.8G110600_v2.0.a1	0.419331754	1.1777727	down
Prupe.8G117300_v2.0.a1	1.370225452	3.871400461	down
Prupe.8G122700_v2.0.a1	0.439408775	0.162925785	up
Prupe.8G127500_v2.0.a1	11.94121071	3.984849901	up
Prupe.8G130200_v2.0.a1	6.526004781	1.540168329	up
Prupe.8G135100_v2.0.a1	5.922022261	2.327128732	up
Prupe.8G136400_v2.0.a1	0	0.903025161	down
Prupe.8G137900_v2.0.a1	1.772385785	5.468935184	down
Prupe.8G146400_v2.0.a1	13.54676227	27.85031956	down
Prupe.8G153700_v2.0.a1	0	1.356981873	down
Prupe.8G157300_v2.0.a1	16.54708874	7.906296085	up
Prupe.8G161600_v2.0.a1	2.586858605	1.294874134	up
Prupe.8G163500_v2.0.a1	151.560303	68.2446042	up
Prupe.8G166400_v2.0.a1	44.68786422	17.83027584	up

Prupe.8G172900_v2.0.a1	49.76986623	119.819002	down
Prupe.8G173100_v2.0.a1	1.099531941	3.140404038	down
Prupe.8G185300_v2.0.a1	0.014188366	0.170450587	down
Prupe.8G192100_v2.0.a1	1.008206449	0.336444224	up
Prupe.8G201000_v2.0.a1	0.851958516	1.0898297	down
Prupe.8G203000_v2.0.a1	0.086372651	0.648518506	down
Prupe.8G210600_v2.0.a1	14.0719848	5.269023144	up
Prupe.8G210700_v2.0.a1	37.25302377	15.25434795	up
Prupe.8G210800_v2.0.a1	94.60035273	41.27486529	up
Prupe.8G219700_v2.0.a1	0.169507748	0.339394195	down
Prupe.8G228500_v2.0.a1	4.657094801	11.92680636	down
Prupe.8G235600_v2.0.a1	2.827275565	1.001007071	up
Prupe.8G239300_v2.0.a1	0.367990076	0.994683081	down
Prupe.8G252500_v2.0.a1	1.401774594	2.40187116	down
Prupe.8G256000_v2.0.a1	0.960614094	0.240421788	up
Prupe.8G256200_v2.0.a1	1.774892451	5.939369557	down
Prupe.8G266100_v2.0.a1	0.350700384	0.902808348	down
Prupe.I000200_v2.0.a1	172.1411131	67.91331971	up

Supplementary Table 2. Primers used in this study.

**Primer ID**	**Sequences (5--3)**	**Application**
PpSnRK1βγ_F1	TGGCAAGGGCCAATTTGTTG	qPCR
PpSnRK1βγ_R1	CAGAGACCAAACGTCGTGGA	
RPll-F	TGAAGCATACACCTATGATGATGAAG	
RPll-R	CTTTGACAGCACCAGTAGATTCC	
Soly_F	TCTTCCGACACCATCGACAA	
Soly_R	AGAACTGCAACACAGTGAGC	
PpSnRK1βγ-CDS-F1	ATGTTTGGTTCTGGTCCGGG	Gene function
PpSnRK1βγ-CDS-R1	CTAGCCTAGCAAGAACCTGAATACAT	
PpSnRK1βγ-CDS-F2	ATGTTTGGTTCTGGTCCGGG	Subcellular localization
PpSnRK1βγ-CDS-R2	GCCTAGCAAGAACCTGAATACAT	
P.LUC.P1.F	gggccccccctcgaggtcgacAAACATTCTCTTTGATGCGTCGA	Promoter activity analysis
P.LUC.P1.R	cgctctagaactagtggatccTATTATAGTGGCAGTAAATTATTTTTTAATGA	
P.LUC.P2+P1.F	gggccccccctcgaggtcgacAAACCAACAACTACTCGTGAGATTGG	
P.LUC.P2+P1.R	cgctctagaactagtggatccTATTATAGTGGCAGTAAATTATTTTTTAATGA	
P.LUC.P3RC+P1.F	gggccccccctcgaggtcgacTTAAATTGGGGCTTCACATAATTTA	
P.LUC.P3RC+P1.R	cgctctagaactagtggatccTATTATAGTGGCAGTAAATTATTTTTTAATGA	
